# Tar spot susceptibility of corn influences phyllosphere-associated bacterial and fungal microbiomes

**DOI:** 10.3389/fmicb.2025.1581312

**Published:** 2025-10-07

**Authors:** Raksha Singh, Wily R. Sic-Hernandez, Charles F. Crane, Sujoung Shim, Darcy E. P. Telenko, Stephen B. Goodwin

**Affiliations:** ^1^Crop Production and Pest Control Research Unit, U.S. Department of Agriculture-Agricultural Research Service (USDA-ARS), West Lafayette, IN, United States; ^2^Department of Botany and Plant Pathology, Purdue University, West Lafayette, IN, United States

**Keywords:** maize, microbial diversity, microbiome, *Phyllachora maydis*, phyllosphere, tar spot

## Abstract

Tar spot, caused by the obligate fungal pathogen *Phyllachora maydis*, is a major economic concern for corn producers in the United States. To test the hypothesis that *P. maydis* can interact with other foliar microorganisms, we investigated phyllosphere microbiomes in relation to corn inbreds with differential tar spot symptoms under natural field infestation. Sixteen inbred lines were assessed for tar spot symptoms, and bacterial and fungal microbiomes were characterized using Illumina MiSeq Sequencing. Comparison of the phyllosphere microbiomes revealed distinct bacterial and fungal communities between resistant and susceptible lines in a sample-specific manner. Bacterial alpha diversity did not differ significantly between susceptible and resistant lines, while fungal diversity (richness, evenness, and phylogenetic diversity) was significantly higher in resistant lines. Beta diversity analysis revealed significant structural differences in both bacterial and fungal communities, with fungal differences more pronounced and driven by relative abundance. Resistant lines were associated with plant-beneficial bacterial genera such as *Quadrisphaera, Klenkia* and *Nocardioides* and fungal genera *Cladosporium, Coniothyrium, Alternaria, Epicoccum, Bipolaris, Phyllozyma*, and *Papiliotrema,* while susceptible lines were dominated by Erwiniaceae, *Aureimonas, Pseudomonas, Microbacterium,* and *Deinococcus* and fungal genera *Hannaella, Paraphaeosphaeria, Sphaerellopsis* and *Phyllachora. Coniothyrium*, a potential mycoparasite of *P. maydis,* was also detected but whether it is the same species that was identified in Central and South America is unknown. Our findings imply that *P. maydis* infection may result in a distinct, less diverse microbiome. Differential abundance analysis revealed enrichment of Erwiniaceae and Enterobacterales, particularly *Amnibacterium* in susceptible lines, and Microbacteriaceae in resistant lines. Correlation analysis between *P. maydis* reads and abundant taxa revealed a predominance of negative correlations, suggesting increased ecological niche differentiation driven by intense competition within the microbial community. These findings suggest that *P. maydis* infection is associated with a less diverse microbiome and that specific microbial taxa may play roles in *P. maydis* susceptibility and resistance. Further research on these correlated and distinct microbiotas could elucidate the role of foliar microbiomes in causing or resisting *P. maydis* infection.

## 1 Introduction

Corn (maize; *Zea mays* subsp. *mays*) is one of the most widely grown crops in the world and is used for a wide range of food, feed and industrial applications. Among numerous risks to worldwide corn production, disease-related yield losses pose a serious threat to our capacity to attain long-term food and nutritional security, and inflict severe financial stresses to growers (Savary et al., 2019). Several foliar diseases, including northern leaf blight (NLB) caused by *Exserohilum turcium* (Hooker, 1976), southern leaf blight (SLB) caused by *Bipolaris maydis* (anamorph: *Cochliobolus heterostrophus*) (Ullstrup, 1972), gray leaf spot caused by *Cercospora zeae-maydis* and *C. zeina* (Tehon and Daniels, 1925; Crous et al., 2006), and common and southern rust caused by *Puccinia sorghi* and *P. polysora* (Cummins, 1941), respectively, can contribute to yield loss.

Another foliar disease, tar spot, has recently emerged as a major threat to corn production, causing yield losses of up to 25–50% on susceptible hybrids under favorable conditions (Rocco da Silva et al., 2021; Valle-Torres et al., 2020). Tar spot is caused by the obligately biotrophic fungal pathogen *Phyllachora maydis* Maubl and was first identified in Indiana and Illinois in 2015 (Bajet et al., 1994; Bissonnette, 2015; Ruhl et al., 2016). After infection, *P. maydis* produces black, glossy, elevated, round or oval stromata on the leaves and stems of corn. In some cases, a fish-eye symptom occurs when stromata are encircled by a tan-colored necrotic halo (Bajet et al., 1994; Hock et al., 1995; McCoy et al., 2019). Although *P. maydis* can infect corn at any stage of growth, the most severe symptoms develop during the silk (R1) and later stages (Singh et al., 2023).

Possible management strategies for tar spot include host resistance, biological control, and fungicidal chemicals. Host resistance should be the most effective, economical and environmentally benign strategy (Rocco da Silva et al., 2021; Valle-Torres et al., 2020) but is not widely available in commercial cultivars. A diverse panel of tropical and subtropical corn germplasm has been screened for tar spot resistance or tolerance with the intent to develop improved varieties (Cao et al., 2017; Lipps et al., 2022; Ren et al., 2022; Singh et al., 2023), but less is known about resistance in North American corn cultivars (Lipps et al., 2022; Singh et al., 2023). Biological control is also being investigated, and several taxa in the corn foliar microbiome have been found to inhibit specific pathogens (Singh and Goodwin, 2022). Thoroughly understanding the microbial response to *P. maydis* infection might reveal microbes associated with resistance that can suppress tar spot disease.

The phyllosphere contains diverse epiphytic and endophytic organisms that can exhibit commensal, parasitic, or mutualistic interactions with leaf tissue. Phyllospheric organisms can significantly affect their host and the ecosystem at large (Lindow and Brandl, 2003). Disease resistance and susceptibility have been linked to the phyllospheric microbiome in a variety of species, including tomato (*Solanum lycopersicum*), poplar (*Populus*), wheat (*Triticum aestivum*), rice (*Oryza sativa*), corn (*Zea mays*), citrus (*Citrus sinensis*) and *Arabidopsis thaliana*, and disease can be altered by manipulating phyllospheric microbes (Berg and Koskella, 2018; Busby et al., 2016; De Costa et al., 2006a, 2006b; Massart et al., 2015; Ritpitakphong et al., 2016; Li P. D. et al., 2022). Despite the importance of phyllospheric microorganisms for plant health, relatively little is known about their response to fungal foliar diseases. Only a few studies have probed the dynamics of microbial populations in the corn phyllosphere in response to fungal pathogens (Balint-Kurti et al., 2010; Manching et al., 2014; McCoy et al., 2019; Singh and Goodwin, 2022; Wagner et al., 2020; Wallace et al., 2018; Ferreira et al., 2025; Sic-Hernandez et al., 2025). For example, particular phyllospheric bacteria may boost resistance to *Cochliobolus heterostrophus* infection (Balint-Kurti et al., 2010). That study also correlated high bacterial diversity with Southern leaf blight (slb) susceptibility in the field. Manching et al. (2014) found that severe slb disease is correlated with decreased species richness of epiphytic bacteria. Phyllospheric fungal communities also have been analyzed in fish-eye and marginless tar spot lesions in corn (McCoy et al., 2019). Recent research has shown that fungal communities in symptomatic tissues where *Phyllachora maydis* dominated were more diverse than those from leaf samples without symptoms, revealing distinct co-occurrence networks involving key genera like *Fusarium* and *Epicoccum*, and confirming low genetic diversity within *P. maydis* populations in Florida (Ferreira et al., 2025).

Despite all these studies, it is not clear whether microbiome structure and disease resistance are directly related or independent processes. Extensive analysis of microbial variation and its correlation to disease, including functional characterization of beneficial microbes, is required to predict and mitigate disease progression. Host disease resistance could be enhanced by the action of antagonist species and/or the net competitiveness of a highly diverse microbiome. Beneficial and/or antagonistic microbes from the corn microbiome may contribute to host resistance by directly inhibiting or suppressing the growth of pathogens or by secreting antimicrobial secondary compounds (Morelli et al., 2020; Singh and Goodwin, 2022; Trivedi et al., 2020).

As mentioned above, the association of phyllosphere community diversity with disease resistance has been studied previously to some extent (Balint-Kurti et al., 2010; Manching et al., 2014; McCoy et al., 2019; Wagner et al., 2020; Wallace et al., 2018). However, connections between the phyllosphere microbiome and tar spot disease severity have never been investigated except for recent analyses showing differences in fungal community structure between symptomatic and asymptomatic corn tissues in Florida (Ferreira et al., 2025). Because the tar spot pathogen exists almost exclusively on the leaf, it and other biotrophic pathogens may be more affected by foliar microbiomes compared to pathogens that live inside host cells. The purpose of this research was to test the hypothesis that microbial community structure differs significantly between resistant and susceptible inbred lines of corn after infection by *P. maydis*. This was accomplished by using Illumina Miseq sequencing to characterize and census the bacteria and fungi associated with resistant and susceptible inbred lines by sequencing the bacterial 16S ribosomal ribonucleic acid (rRNA) gene and the fungal 18S rRNA gene and internal transcribed spacer (ITS) region. We also revealed the significant differential abundance of bacterial and fungal taxa between resistant and susceptible inbred lines. A thorough understanding of microbial diversity and its correlation to disease between resistant and susceptible inbred lines is potentially useful to identify microbes that affect disease progression. Overall, our findings reveal significant differences in community richness and abundance of bacterial and fungal taxa between resistant and susceptible inbred lines of corn after infection by *P. maydis*.

## 2 Materials and methods

### 2.1 Corn lines and growth conditions

In this study, we analyzed the phyllosphere microbiome of 16 inbred corn lines that differed in severity of tar spot after the onset of symptoms under natural infection in field conditions. Among 16 inbred lines, five were parental lines of the Nested Association Mapping (NAM) population (CML52, CML69, CML103, TX303 and B97) (McMullen et al., 2009; Yu et al., 2008) and the remaining 11 lines (PI685788, PI658790, PI685806, PI685831, PI685836, PI685915, PI685918, PI685919, PI685920, PI685950, and 4,401,350) were from the Germplasm Enhancement of Maize (GEM) project (Pollak and Salhuana, 2001). Corn inbred lines were planted in three replicates at a research field trial located at the Pinney Purdue Agricultural Center, Wanatah, Indiana. This location was chosen for the experiment because of high tar spot disease pressure detected in prior years. Because of its obligately biotrophic nature, *P. maydis* cannot be cultured for inoculation, hence all field studies relied on natural infection. Plots were planted on June 8, 2019, with approximately 20 seeds per row with two rows for each line in a randomized complete block design (RCBD) with three replications. Experimental plots were 3.0 m (10ft) wide and 9.1 m (30ft) long, consisting of two rows for disease evaluation. At all locations, the previous crop was corn with a history of tar spot. Fungicide treatments were not applied during the experimental period to allow for natural infection. After the onset of tar spot symptoms, scoring was conducted at the late dent reproductive growth stage (R5) in early October and was conducted as described previously (Singh et al., 2023). This 0-to-100% disease severity scale at different growth stages has been used as a standard in numerous prior publications (Kleczewski and Bowman, 2020; Telenko et al., 2019, 2022; Singh et al., 2023). Four plants were rated from each line in two replicates, beginning 2 weeks after the first observed disease incidence. To capture whole-plant disease severity, ratings were taken from three parts of the canopy: lower leaf (defined as two leaves below the ear leaf), ear leaf, and upper leaf (defined as two leaves above the ear leaf). The means of these four ratings were used for calculating the disease severity for each plant.

### 2.2 Sample collection and processing

Leaves from corn lines that ranged from susceptible to tolerant to *Phyllachora maydis* were collected at 14 weeks after planting when plants were in the late dent reproductive growth stage (R5). In total, 48 samples were collected from 16 inbred lines including three replicates. For each replicate, five ear leaves were sampled. Sampled leaves were sprayed and wiped with 70% ethanol, and then dried between sterile paper towels. Six leaf discs uniformly spaced from the base to the tip of each leaf were excised using a sterilized cork borer (18 mm), avoiding the midrib and any dry tissue, so that each replication consisted of 30 leaf discs from 5 individual leaves. Excised leaf discs were immediately transferred to sterile microcentrifuge tubes using sterilized forceps and were stored at −80 °C until DNA extraction.

### 2.3 DNA extraction

DNA was extracted using the Synergy 2.0 Plant DNA Extraction Kit (OPS Diagnostics, Lebanon, NJ, USA) with some modifications. Briefly, leaf discs were ground in liquid nitrogen and homogenized with 500 μL of Plant Homogenization Buffer with a bead beater at its highest speed for 1 min. Homogenates were centrifuged at 15,000 × *g* for 5 min at room temperature to pellet debris. Clear supernatant was transferred to a sterilized microcentrifuge tube and RNA was removed by adding 5 μL of RNase A solution, briefly vortexing to mix and incubating at 37 °C for 15 min. The extracted DNA was precipitated with isopropanol and transferred to a silica spin column followed by centrifugation at 8,000 × *g* for 1 min. The DNA samples were washed twice with 250 μL of ice-cold 70% ethanol followed by centrifugation at 8,000 × g for 1 min and dissolved in 50 μL of molecular biology-grade water.

### 2.4 MetaVx™ library preparation and Illumina MiSeq sequencing

Library preparations and Illumina MiSeq sequencing were conducted at GENEWIZ, Inc. (South Plainfield, NJ, USA). DNA samples were quantified using a Qubit 2.0 Fluorometer (Invitrogen, Carlsbad, CA, USA) and 30–50 ng of DNA were used to generate amplicons using a MetaVx™ Library Preparation kit (GENEWIZ, Inc., South Plainfield, NJ, USA).

### 2.5 16S method for bacteria

For bacterial microbiomes the V3, V4, and V5 hypervariable regions of prokaryotic 16S rDNA were selected for generating amplicons and subsequent taxonomy analysis. GENEWIZ designed a panel of proprietary primers aimed at relatively conserved regions bordering the V3, V4, and V5 hypervariable regions of bacteria and Archaea 16S rDNA. The V3 and V4 regions were amplified using forward primers containing the sequence (5′ to 3′) “CCTACGGRRBGCASCAGKVRVGAAT” and reverse primers containing the sequence “GGACTACNVGGGTWTCTAATCC.” The V4 and V5 regions were amplified using forward primers containing the sequence “GTGYCAGCMGCCGCGGTAA” and reverse primers containing the sequence “CTTGTGCGGKCCCCCGYCAATTC.” First-round PCR products were used as templates for second-round amplicon-enrichment PCR. At the same time, adapters were added to the ends of the 16S rDNA amplicons to generate indexed libraries ready for downstream NGS sequencing on an Illumina Miseq machine.

### 2.6 ITS method for fungi

From 50 to 100 ng of DNA were used to generate amplicons using a panel of primers designed by GENEWIZ (GENEWIZ, Inc., South Plainfield, NJ, USA). Multiple oligonucleotide primers were designed to anneal to the relatively conserved sequences spanning fungal ITS regions. The ITS2 region was amplified using a forward primer containing sequence “GTGAATCATCGARTC” and reverse primer containing sequence “TCCTCCGCTTATTGAT.” In addition to the ITS target-specific sequences, the primers also contain adaptor sequences allowing uniform amplification of the library with high complexity ready for downstream NGS sequencing on the Illumina Miseq platform.

DNA libraries were validated with an Agilent 2,100 Bioanalyzer (Agilent Technologies, Palo Alto, CA, USA), and quantified using a Qubit 2.0 Fluorometer. DNA libraries were multiplexed and loaded on an Illumina MiSeq instrument according to the manufacturer’s instructions (Illumina, San Diego, CA, USA). Sequencing was performed using a 2×300/250 paired-end (PE) configuration; image analysis and base calling were conducted by the MiSeq Control Software (MCS) embedded in the MiSeq instrument.

### 2.7 Data processing and analysis: construction of a database for microbial rDNA sequences

For fungi, the UNITE ITS database (Released on 07-18-2023) appended with sequences of *Cladosporium* (*n* = 4), *Epicoccum nigrum* (*n* = 5) and *Alternaria* (*n* = 3) was used for fungal classification. Files were transformed to Qiime2 artifacts and trained using the naive Bayes classifier method (Bolyen et al., 2019). For bacteria, SILVA v138.1 taxonomy files were retrieved and transformed to Qiime2 artifacts, then used to generate a SILVA fixed-rank taxonomy artifact. The non-redundant and unaligned (NR99) sequence file was also retrieved from the SILVA database and transformed to a Qiime2 artifact, then sequences were converted from RNA to DNA. Sequences containing five or more ambiguous bases were removed from the artifact file. Next, reference sequences were filtered by length and taxonomy: Bacterial (16S) sequences shorter than 1,200 bp, Archaeal (16S) sequences shorter than 900 bp, and Eukaryotic (18S) sequences shorter than 1,400 bp were removed. Redundant sequences were removed and only sequences with unique taxonomic affiliations were retained. The V3 to V4 amplicon region was extracted from the sequence file and used to create a classifier using the naïve Bayes classifier method.

### 2.8 Quality filtering

Quality-filtered ribosomal reads were obtained from Genewiz, Inc. (South Plainfield, NJ, USA). There were separate forward and reverse FASTQC files for each inbred. These FASTQ files were analyzed using Quantitative Insight into Microbial Ecology (QIIME2) v.2024.2. Using DADA2 (Callahan et al., 2016), which involved denoising, dereplicating and removing chimeras; fungal sequences were trimmed after 22 base pairs for forward reads and 21 base pairs for reverse reads, with truncation set to 190 base pairs for both to ensure that the sequences had a quality greater than Q20. Bacterial sequences were trimmed after 21 base pairs for forward reads and 23 base pairs for reverse reads and truncated to 248 and 233 base pairs for forward and reverse reads, respectively, to achieve the same quality threshold (Q > 20). DADA2 subsequently clustered the sequences into amplicon sequence variants (ASVs) with single-nucleotide resolution.

### 2.9 Taxonomic annotation and relative abundance analysis

ASVs were annotated with QIIME2 using the fitted classifier Sklearn and database classifiers previously created for fungi and bacteria. Sequences annotated as mitochondrial or chloroplast were removed for both fungal and bacterial datasets. Then sequences were rarified to 67,200 sequences per sample for fungi and 13,700 sequences per sample for bacteria. QIIME2 outputs were analyzed in R using the package “phyloseq” version 1.48.0. (McMurdie and Holmes, 2013) to create bar plots using the package “ggplot2” version 3.5.1 (Wickham, 2016). In R analysis if an ASV was not classified at a higher level (e.g., species), it was retained and assigned to the lowest taxonomic level for which classification was available (e.g., genus) to ensure it was not excluded from the analysis. As a result, family or genus bar plots may show taxon labels such as “uncl. Pleosporales,” which stands for unclassified Pleosporales. For both fungal and bacterial families, genera and species were filtered to show only the 30 most abundant; as a result bars do not reach the 100% of relative abundance.

### 2.10 Population diversity analysis (alpha and beta diversity)

Alpha and beta diversities were estimated in QIIME2 using the Faith’s PD (Faith, 1992), Shannon (Shannon, 1948) and Pielou evenness (Pielou, 1966) indexes for alpha and Bray-Curtis’ dissimilarity (Bray and Curtis, 1957) and Unweighted UniFrac (Lozupone and Knight, 2005) indexes for beta diversity. Alpha diversity indexes were used to compare the diversity between corn inbreds while beta diversity indexes were used to compare resistant and susceptible corn inbreds. Statistical analysis between resistant and susceptible corn inbred lines was performed by the QIIME2 plug in “diversity,” which performed a permutational multivariate analysis of variance test (PERMANOVA) of the Bray-Curtis and Unweighted UniFrac indexes. Statistical analysis between corn inbred lines also was obtained with Dunn’s test [results adjusted with the Benjamini and Hochberg method (BH)]. QIIME2 outputs were analyzed in R using the package “phyloseq” version 1.48.0 (McMurdie and Holmes, 2013) to create box plots using the package “ggplot2” version 3.5.1 (Wickham, 2016) for alpha indexes and principal coordinate analysis (PCoA) plots for beta indexes.

### 2.11 Differential abundance analysis between resistant and susceptible corn inbred lines

The Analysis of Compositions of Microbiomes with Bias Correction (ANCOM-BC) algorithm (Lin and Peddada, 2020) was used to identify species that are differentially abundant between resistant and susceptible corn inbred lines. The analysis was conducted in QIIME2 using the plugin “q2-composition” version 2024.2.0. Bacterial species with relative abundance of less than 0.01% were discarded. The threshold for significance was set to a Benjamini–Hochberg false discovery rate-corrected *p* value of < 0.05. Species with significant differential abundance (*p* ≤ 0.05) between resistant and susceptible lines were presented in a bar plot.

### 2.12 Correlation analyses

Correlations between *P. maydis* and the 20 most abundant fungal or bacterial species were analyzed in R using Spearman’s rank correlation coefficients, calculated with the cor() function. The statistical significance of the correlation matrix was assessed using the cor.mtest() function from the corrplot package, with a 95% confidence level. Pearson correlation coefficients between the relative frequencies of 20 most-abundant bacterial and fungal taxa with total counts of *P. maydis* reads were calculated by using the R function cor.test. The ASVs with the highest sequence read counts of the 20 most-abundant bacterial and fungal taxa were compared to sequences in the NCBI core nucleotide database to determine the percentage of identity and nucleotide differences. To identify species, we reported the species with the highest number of hits, even when multiple species within the same genus shared identical e-values, query coverages, and percentage identities. The objective was to find any significant change in the top 20 taxa that could indicate a response other than simple, physical displacement of other taxa by *P. maydis*.

## 3 Results

### 3.1 Inbred corn lines that varied for tar spot severity under natural infection were used for Miseq sequencing

Disease scoring data based on a previously published protocol as mentioned in the Materials and Methods showed that the inbreds PI685918 and TX303 were susceptible; inbreds PI685920, 4401350, B97, PI685836, and PI685915 were moderately susceptible; inbreds B97, PI685790, and PI685831 were moderately resistant; and inbreds PI685788, PI685950, PI685919, PI685806, CML103, CML69, and CML52 were resistant (Table 1). For downstream microbiome analysis, these categories were simplified: moderately susceptible and susceptible were combined into “susceptible,” and moderately resistant and resistant into “resistant.” As a result, inbreds PI685918, TX303, PI685920, 4401350, PI685836, and PI685915 were classified as susceptible, while inbreds B97, PI685790, PI685831, PI685788, PI685950, PI685919, PI685806, CML103, CML69, and CML52 were classified as resistant (Table 1). Representative images illustrate clear differences between resistant and susceptible corn lines (Supplementary Figure S1).

**Table 1:** Disease scores and percent *Phyllachora maydis* sequence reads for 16 inbred lines of corn (*Zea mays s*sp. *mays*) chosen from the parents of the Nested Association Mapping (NAM) and Germplasm Enhancement of Maize (GEM) genetic populations.

Corn inbred line	Mean severity rating^1^	Disease phenotype^2^	Percent *Phyllachora maydis* reads	Disease status combining phenotypic and Miseq data
PI685920	28.46	MS	92.35	S
B97	7.40	MR	89.23	S
PI685788	3.30	R	87.97	S
4401350	27.66	MS	86.85	S
PI685918	34.33	S	84.39	S
PI685836	22.46	MS	80.0	S
PI685950	1.14	R	76.76	S
PI685919	0.23	R	73.76	S
PI685790	10.56	MR	69.18	S
PI685915	14.03	MS	65.73	S
PI685806	0.20	R	26.99	R
PI685831	10.53	MR	23.39	R
CML69	0.00	R	15.05	R
CML103	0.34	R	9.39	R
CML52	0.22	R	0.00*	R
TX303	36.0	S	0.00*	S

^1^Mean tar spot severity (% of stromata) was scored for three replicates of 16 corn inbred lines located at the Pinney Purdue Agricultural Center, Wanatah, Indiana. ^2^Disease phenotype based in accordance with Singh et al. (2023). R: Disease phenotype ranged from 0 to 5%; MR: Disease phenotype ranged from >5 to 11%; MS: Disease phenotype ranged from >11 to 30%; S: Disease phenotype ranged from >30% (Singh et al., 2023). *Percent *P. maydis* reads in CML52 and TX303 were not detected even though disease symptoms were detected visually because the sequencing reads are of low quality so that reverse and forward reads did not overlap to pass the quality filters for QIIME analysis.

However, MiSeq sequencing revealed discrepancies between visual scoring and *P. maydis* read counts. Several lines initially classified as resistant based on visual scores (PI685950, PI685919, PI685788, PI685790, and B97) contained over 30% *P. maydis* reads (Table 1). Therefore, a combined analysis of visual and MiSeq data led to a reclassification with only PI685806, PI685831, CML103, CML52, and CML69 ultimately being considered resistant, while the remaining 11 lines were classified as susceptible. Notably, two lines, CML52 and TX303, presented a different discrepancy: while visual disease symptoms were observed, *P. maydis* sequence reads were not detected (0.00) (Table 1). This discrepancy was due to low-quality reverse sequencing reads that did not pass the quality filters during QIIME analysis, which prevented the necessary overlap with forward reads. As a result, the entire read pair (both forward and reverse) may have been discarded, causing the removal of those taxa from further analyses.

Samples PI685950 and TX303 were excluded from bacterial microbiome analysis due to having fewer than 5,000 MiSeq bacterial reads, below the 10,000-read threshold for this study (Supplementary Figure S2). Similarly, TX303 was excluded from further fungal microbiome analysis as it had fewer than 5,000 fungal reads (Supplementary Figure S3). To avoid potential bias from a low number of reads, PI685950 and TX303 were excluded from further bacterial microbiome analysis, and CML52 and TX303 were excluded from further fungal microbiome analysis. Finally, for downstream bacterial microbiome analysis, resistant inbred lines PI685806, PI685831, CML103, CML52, and CML69 and susceptible inbred lines PI685915, PI685790, PI685919, PI685836, PI685918, B97, 4401350, PI685788, and PI685920 were considered. For fungal microbiome analysis, resistant inbreds were PI685806, PI685831, CML103, and CML69, and susceptible inbreds were PI685915, PI685790, PI685919, PI685950, PI685836, PI685918, B97, 4,401,350, PI685788, and PI685920 (Table 1).

### 3.2 Alpha (*α*) and beta (*β*) diversities showed differences in bacterial and fungal communities between resistant and susceptible corn lines

**Figure 1:**
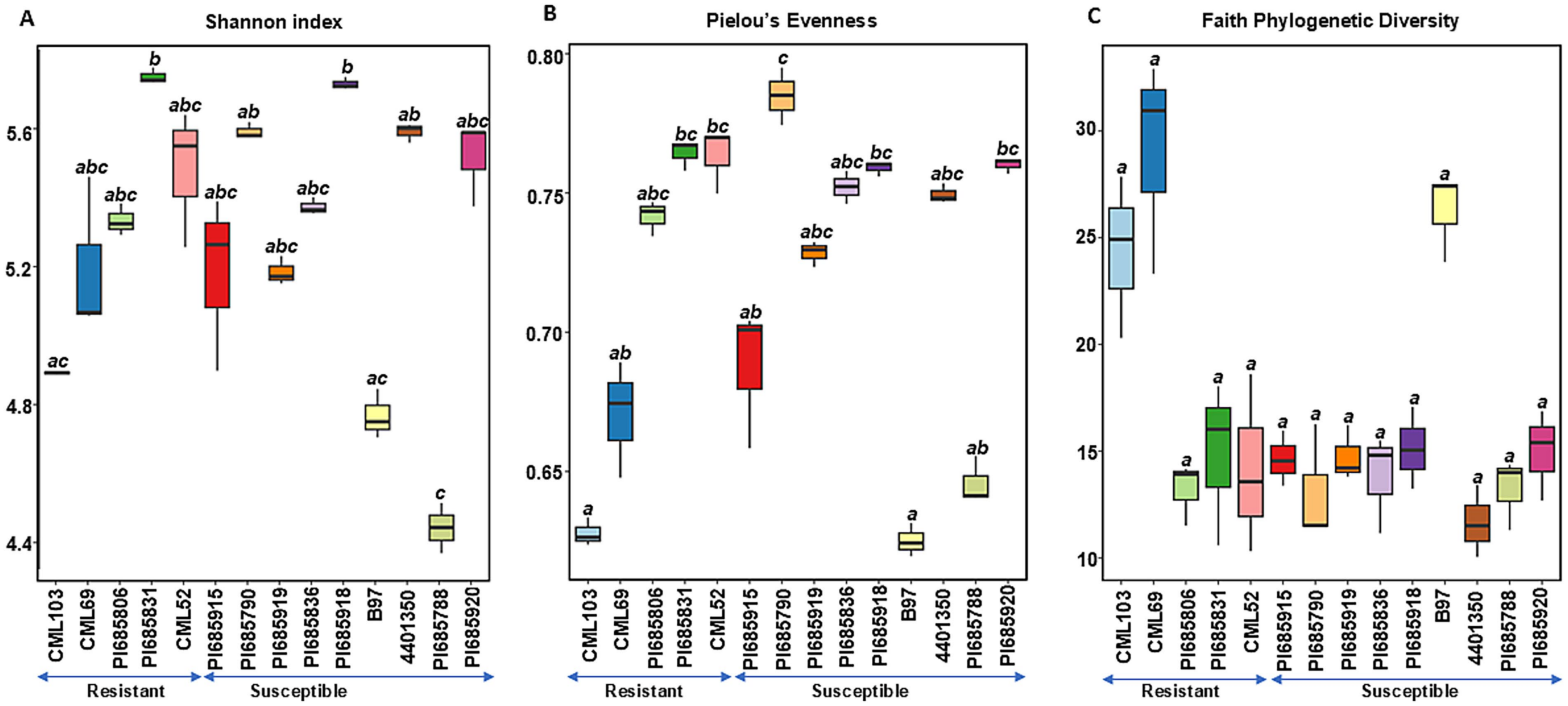
Comparisons of bacterial alpha diversity indices **(A)** Shannon diversity, **(B)** Pielous’s Evenness and **(C)** Faith’s Phylogenetic Diversity observed in *Phyllachora maydis* resistant and susceptible corn lines. Significant differences between corn lines were assessed in pairwise comparisons using Dunn’s test following a non-parametric Kruskal–Wallis rank sum test. *p*-values were adjusted using the Benjamini-Hochberg (BH) method. A statistically significant difference was defined as a BH-adjusted *p*-value ≤ 0.05 (Supplementary Table S2).

**Figure 2:**
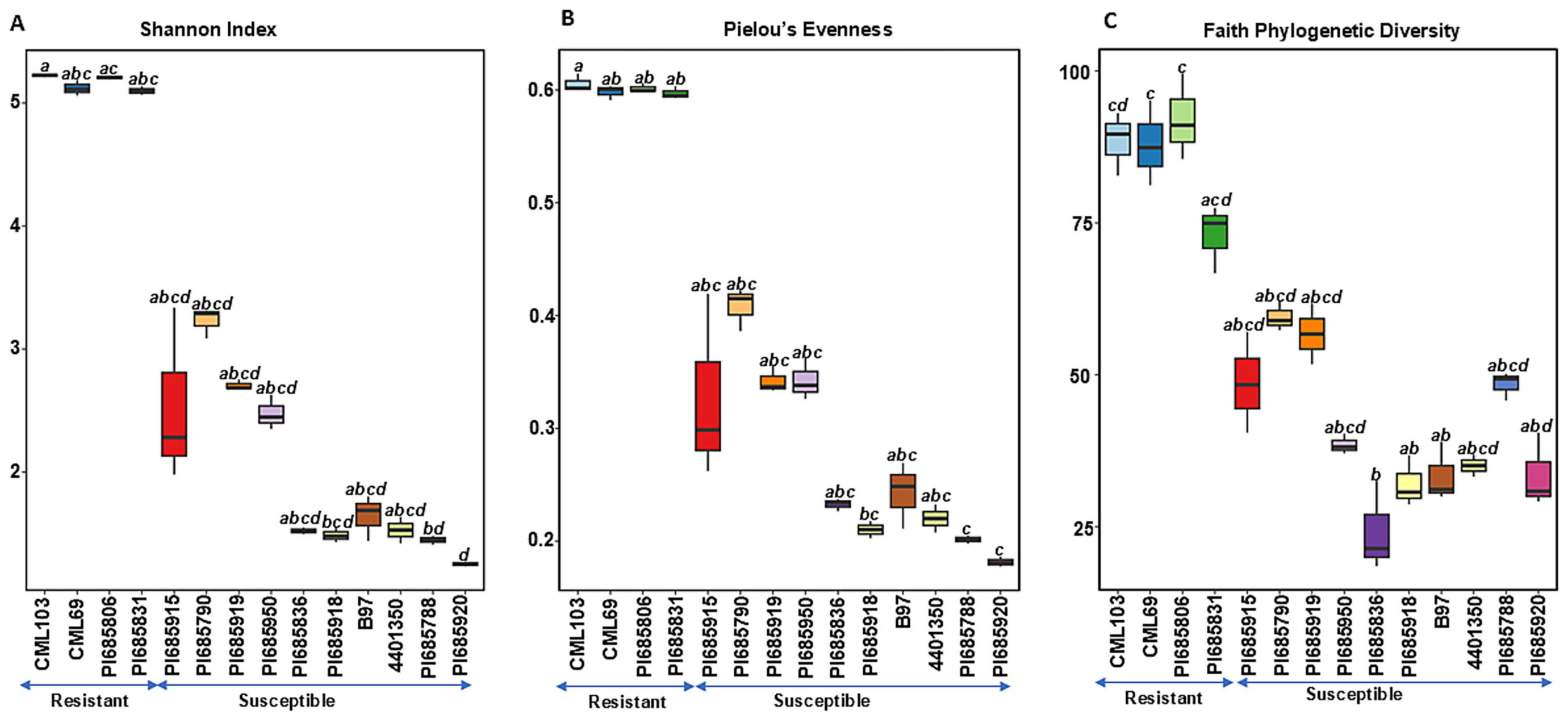
Comparisons of fungal alpha diversity indices **(A)** Shannon diversity, **(B)** Pielous’s Evenness and **(C)** Faith’s Phylogenetic Diversity observed in *Phyllachora maydis* resistant and susceptible corn lines. Significant differences between corn lines were assessed in pairwise comparisons using Dunn’s test following a non-parametric Kruskal–Wallis rank sum test. *p*-values were adjusted using the Benjamini-Hochberg (BH) method. A statistically significant difference was defined as a BH-adjusted *p*-value ≤ 0.05 (Supplementary Table S2).

When compared, resistant and susceptible corn lines showed some variation in alpha diversity for both bacterial and fungal communities; however, the patterns differed depending on the specific sample, the specific diversity metric, and the community type (Figures 1, 2).

For bacterial communities, there was no clear difference in diversity between the resistant and susceptible lines as shown by the Shannon and Pielou’s evenness indices (Figures 1A,B). Although some susceptible inbred lines (PI685788, 4401350, and PI685790) showed significant variation (*p* < 0.05, Supplementary Table S3), the remaining six lines exhibited similar levels of bacterial richness and evenness, suggesting a weak correlation between disease status and richness and evenness (Figures 1A,B). Notably, the resistant line PI685831 and susceptible line PI685918 displayed the highest Shannon diversities among all samples. While most other resistant and susceptible lines exhibited relatively the same level of Shannon diversity, susceptible lines, such as B97 and PI685788, showed notably lower Shannon diversities. For Faith phylogenetic diversity, while susceptible lines tended to have lower values compared to the resistant lines CML103 and CML69, the differences were not statistically significant across all lines tested. Interestingly, resistant lines PI685806, PI685831 and CML52 did not show a clear difference in Faith Phylogenetic diversity compared to the eight susceptible lines (Figure 1C).

In contrast to bacterial communities, fungal communities exhibited significantly higher richness, evenness, and phylogenetic diversity in the resistant lines CML103, CML69, PI685806, and PI685831 compared to ten of the susceptible lines (PI685915, PI685790, PI685919, PI685950, PI685836, PI685918, B97, 4401350, PI685788, and PI685920) (Figures 2A–C). Faith phylogenetic diversity of fungal communities was also significantly lower in ten of the susceptible lines (PI685915, PI685790, PI685919, PI685950, PI685836, PI685918, B97, 4401350, PI685788, and PI685920) compared to the four resistant lines CML103, CML69, PI685806, and PI685831 (Figure 2C). These results indicate a clear difference in fungal community diversity between resistant and susceptible inbreds, with resistant lines CML103, CML69, PI685806, and PI685831 exhibiting greater richness, evenness, and phylogenetic diversity. However, no significant differences in richness, evenness and phylogeny of bacterial communities were observed between the resistant and susceptible lines, except for two susceptible lines, B97 and PI685788, that showed significantly lower richness and evenness (*p* < 0.05, Supplementary Table S3).

**Figure 3:**
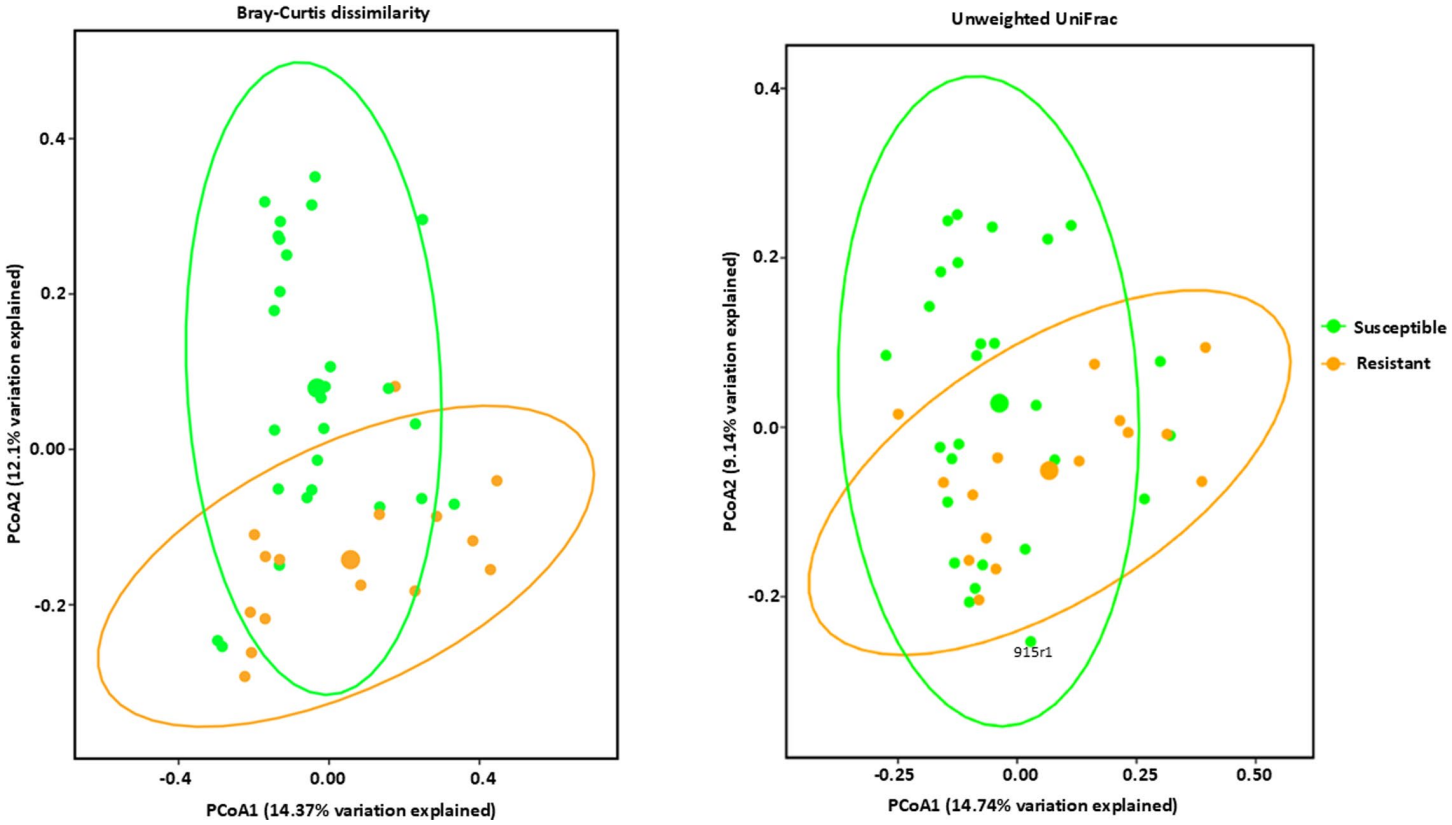
Beta diversity analysis of bacterial communities by PCoA (Principal Coordinate Analysis) with Bray–Curtis dissimilarity values and Unweighted Unifrac observed in *Phyllachora maydis* resistant and susceptible corn lines. Color denotes disease severity pattern (resistant: yellow; susceptible: green). Bacterial communities that are associated with resistant corn lines were displayed as yellow dots and bacterial communities that are associated with susceptible corn lines were displayed as green dots. Ellipses are drawn at 95% confidence intervals for each disease severity pattern. Significant differences between corn lines were calculated by PERMANOVA with 999 permutations (*p*-value ≤0.05) and presented in Supplementary Table S3.

Estimates of beta diversity enable comparisons among microbial communities, considering both the number of distinct microorganisms in the sample and their phylogenetic relatedness, i.e., differences in their specific species compositions. The beta diversities of the microbial communities between the resistant and susceptible lines were compared using permutational multivariate analysis of variance (PERMANOVA) (Anderson, 2001) with the Bray–Curtis dissimilarity (Bray and Curtis, 1957) and Unweighted UniFrac distance metrics. These analyses were visualized through Principal Coordinates Analysis (PCoA) plots, offering complementary perspectives on community dissimilarities. The Bray-Curtis PCoA, which emphasizes differences in species relative abundances, revealed a clear separation between the resistant and susceptible groups that are statistically significant (Figure 3 and Supplementary Table S3). Resistant inbred lines clustered predominantly in the lower right quadrant, indicating a distinct community composition. Susceptible lines were distributed more broadly, mainly in the upper portion of the plot, suggesting a greater degree of variability in their microbial communities (Figure 3). However, the observed overlap, particularly in the center of the ordination space, indicates that some samples share similar community profiles regardless of resistance classification. This overlap could be attributed to several factors, including inherent biological variation, environmental influences not accounted for in the analysis, or the presence of other factors influencing community composition independently of resistance. The UnWeighted UniFrac PCoA, incorporating phylogenetic information and emphasizing differences in lineage presence or absence, corroborated the findings from the Bray-Curtis analysis, revealing a similar clustering pattern but with some key differences (Figure 3 and Supplementary Table S3). While resistant lines still clustered predominantly in the lower right quadrant and susceptible lines were more broadly distributed, the separation between the two groups was less pronounced compared to the Bray-Curtis ordination. This suggests that differences in community membership (i.e., which taxa are present) between resistant and susceptible lines are less distinct than differences in relative abundance. The greater degree of intermingling between groups in this ordination indicates that while resistant and susceptible lines share many of the same taxa, the proportions of these shared taxa differ substantially, as highlighted by the Bray-Curtis analysis.

**Figure 4:**
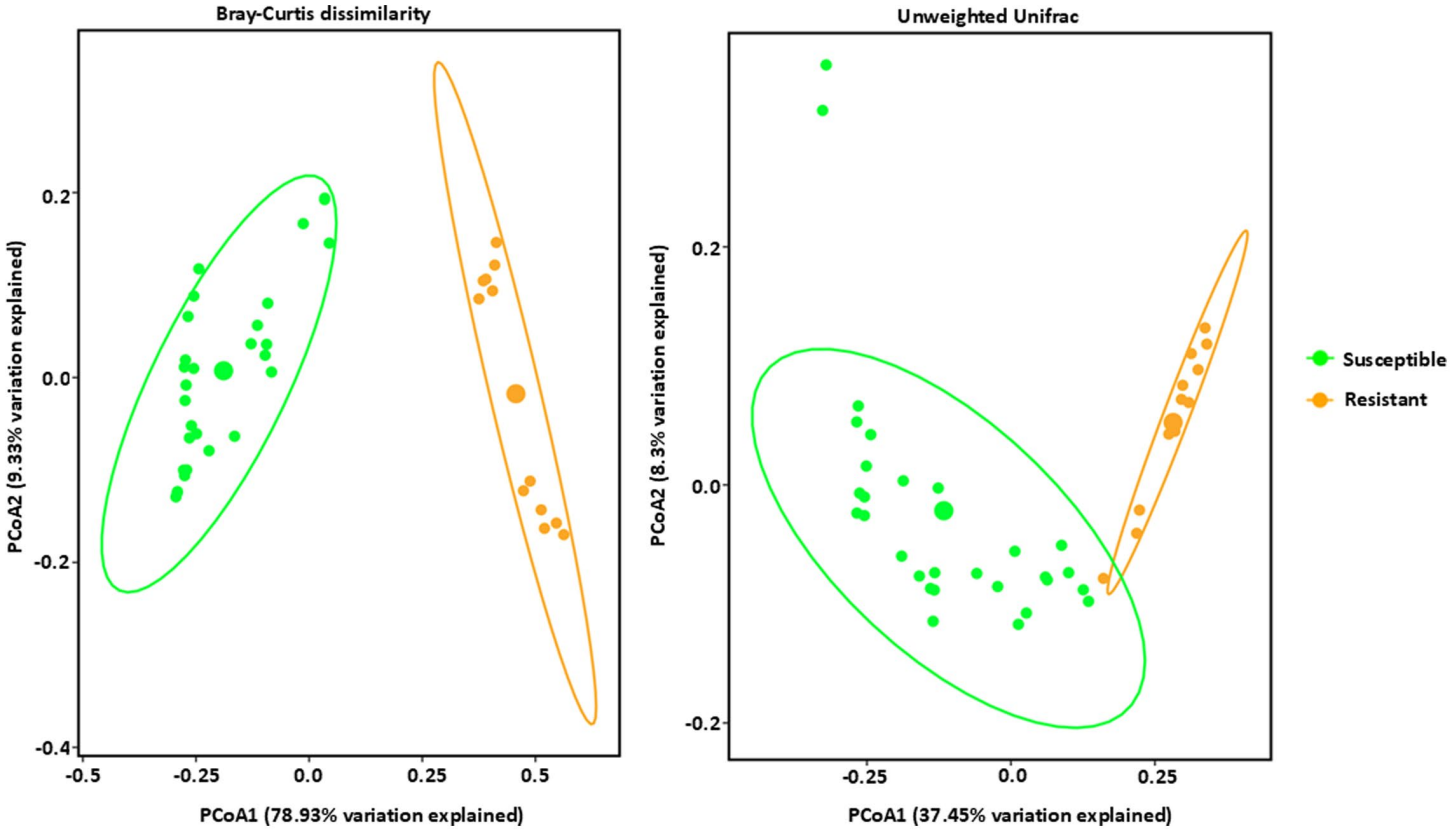
Beta diversity analysis of fungal communities by PCoA (Principal Coordinate Analysis) with Bray–Curtis dissimilarity values and Unweighted Unifrac observed in *Phyllahora maydis* resistant and susceptible corn lines. Color denotes disease severity pattern (resistant: yellow; susceptible: green). Fungal communities that are associated with resistant corn lines were displayed as yellow dots and fungal communities that are associated with susceptible corn lines were displayed as green dots. Ellipses are drawn at 95% confidence intervals for each disease severity pattern. Significant differences between corn lines were calculated by PERMANOVA with 999 permutations (*p*-value ≤ 0.05) and presented in Supplementary Table S3.

In contrast to the bacteria, fungal communities showed significant, distinct clustering between the resistant and susceptible lines (Figure 4 and Supplementary Table S3). All four resistant lines clustered together in a group and the ten susceptible lines clustered together in a different group, showing a distinct clustering from the resistant lines using the Bray–Curtis dissimilarity metric and Weighted Unifrac matrices (Figure 4). The high percentage of variation explained by PCoA1 in Bray–Curtis dissimilarity, indicates that this axis strongly captures the differences in relative abundance driving the separation between resistant and susceptible lines. Susceptible lines were more dispersed, primarily located on the left side of the plot, suggesting a greater degree of variability in their microbial communities. However, Unweighted Unifrac analysis, which emphasizes phylogenetic distances between communities, showed a similar trend but with some key differences. While resistant lines still clustered predominantly in the right side of the ordination space and susceptible lines were more broadly distributed, the separation between the two groups was less pronounced compared to the Bray-Curtis ordination (Figure 4). This suggests that while phylogenetic distances contribute to differences in fungal communities, relative abundance is the dominant factor. In conclusion, these results suggest that tar spot disease susceptibility significantly influences the structure and composition of fungal communities, with relative abundance playing a key role in these differences. Bacterial communities also showed some influence, though less pronounced.

### 3.3 Structure and diversity of the bacterial and fungal communities in resistant and susceptible lines

**Figure 5:**
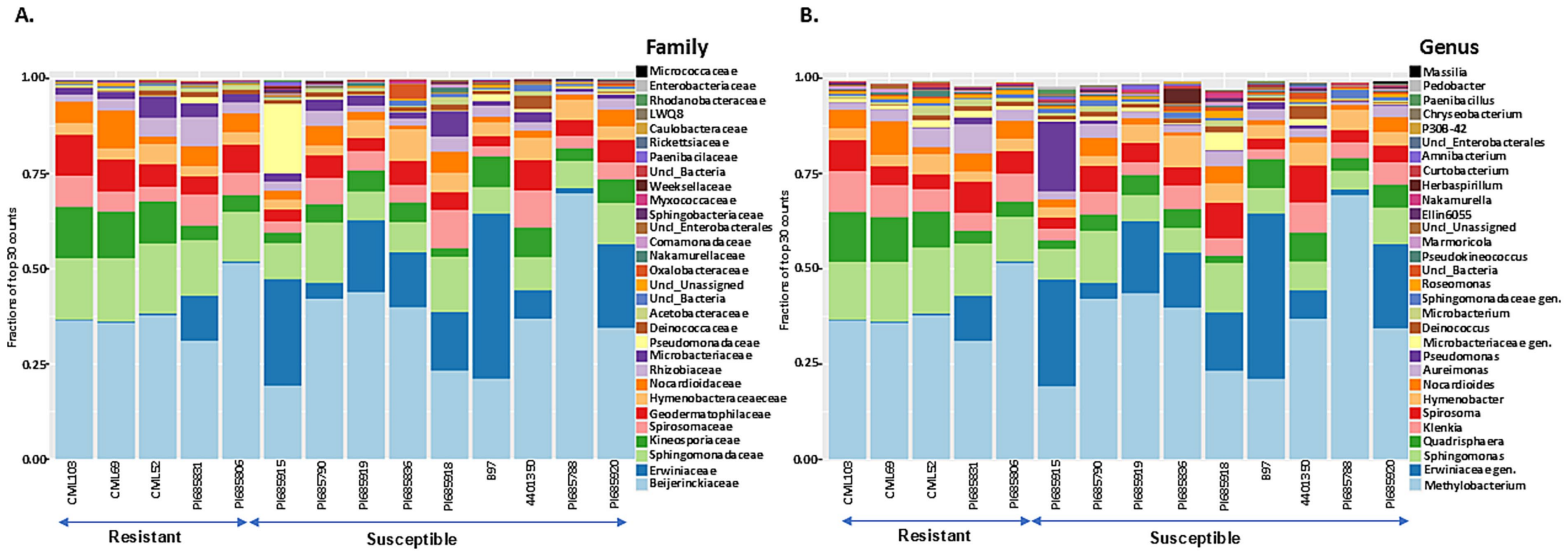
The 30 most abundant bacterial families **(A)** and genera **(B)** in *Phyllachora maydis* resistant and susceptible corn lines. The values represent relative mean abundances (*n* = 3), and each color specifies a listed taxon.

**Figure 6:**
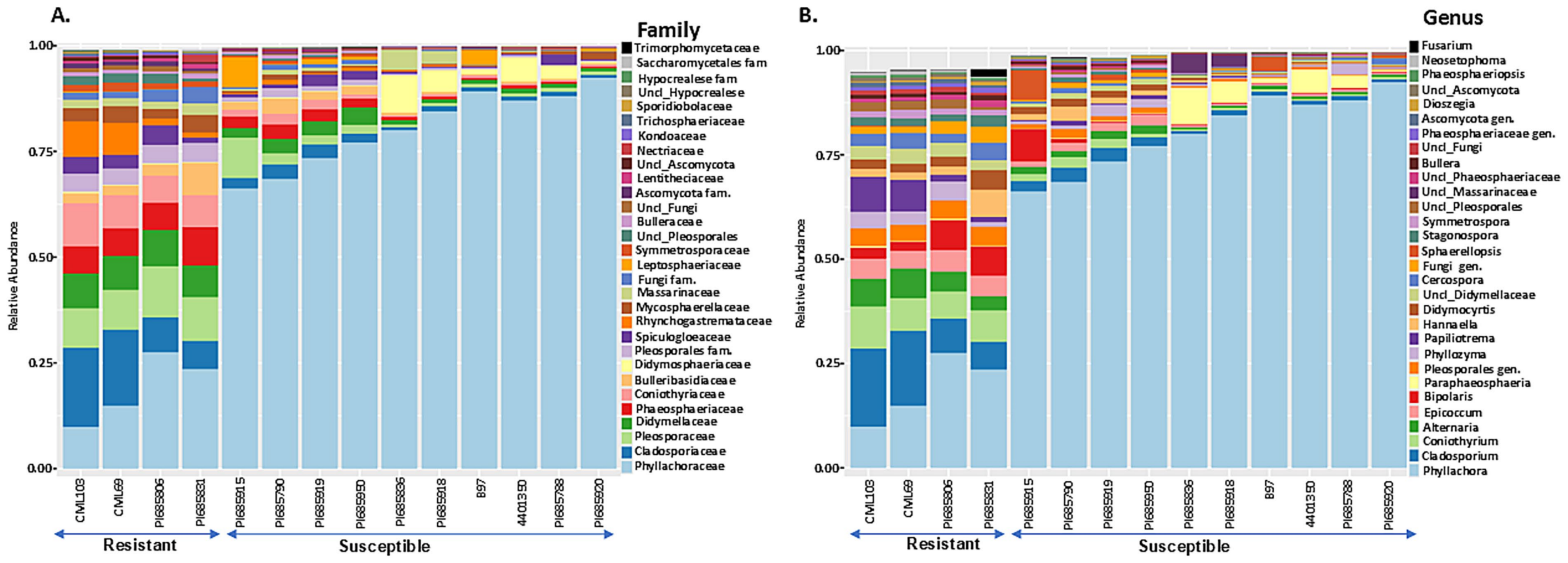
The 30 most abundant fungal families **(A)** and genera **(B)** in *Phyllachora maydis* resistant and susceptible corn lines. The bars represent the relative mean abundances (*n* = 3), and each color specifies a listed taxon.

Taxonomic profiling was performed by mapping bacterial and fungal reads to the reference databases (SILVA 16S v.8.3 for bacterial reads and UNITE fungal ITS v138.1 for fungal reads) to analyze the composition and diversity of microbial communities in tar spot-resistant and -susceptible samples. The Illumina Miseq sequencing of 45 samples including 3 replicates resulted in 10,868,490 bacterial reads and 16,217,432 fungal reads, with on average 113,213 bacterial reads per sample and 172,525 fungal reads per sample. In total, the reads could be characterized into 157 bacterial and 338 fungal genera across all corn lines (Table 2). The analysis of 16S rRNA and ITS region sequencing analyses revealed distinct differences in both bacterial (Figures 5A,B) and fungal (Figures 6A,B) community composition associated with *P. maydis* resistant and susceptible lines. The dominant bacterial families were Beijerinckiaceae in all inbred lines (Figure 5A). Additional families such as Erwiniaceae, Sphingomonadaceae, Kineosporiaceae, Spirosomaceae, Geodermatophilaceae, Hymenobacteraceae, Nocardioidaceae, Rhizobiaceae, Microbacteriaceae, Pseudomonadaceae and Deinococcaceae were also detected to some extent in all inbred lines (Figure 5A). Interestingly, a higher relative abundance of families Erwiniaceae, Spirosomaceae, Rhizobiaceae, Pseudomonaceae, and Deinococcaceae were detected in susceptible lines, while Sphingomonadaceae, Kineosporiaceae and Nocardioidaceae were more prevalent in resistant lines (Figure 5A). At the genus level, dominant bacterial genera were *Methylobacterium*, Erwiniaceae gen., and *Sphingomonas* (Figure 5B). Genera *Methylobacterium*, *Erwiniaceae* gen, *Pseudomonas*, and *Deinococcus* were highly abundant on most of the susceptible lines whereas *Sphingomonas, Quadrisphaera, Klenkia, Nocardiodes, Aureimonas, Microbacterium, Roseomonas* and *Pseudokineococcus* were abundant on resistant lines (Figure 5B). At the phylum level, Proteobacteria, Actinobacteriota, and Bacteroidota were the dominant bacterial phyla on all lines (Supplementary Figure S4).

**Table 2:** Numbers of prokaryotic and eukaryotic taxa that appeared in at least one sample of microbiome sequences from 14 inbred lines of corn that differed in resistance to tar spot caused by the fungal pathogen *Phyllachora maydis*, by taxonomic level.

Taxonomic level	Prokaryotic taxa	Eukaryotic taxa
Phylum	19	5
Class	29	27
Order	63	81
Family	101	200
Genus	157	338

The most abundant fungal families were Phyllachoraceae, Cladosporiaceae, Pleosporaceae, and Didymellaceae, while Coniothyriaceae, Phaeosphaeriaceae, Bulleribasidiaceae, Spiculogloeaceae, and Massarinceae were also detected in lower numbers (Figure 6A). Interestingly, families Cladosporiaceae, Pleosporaceae, Didymellaceae, Phaeosphaeriaceae, Coniothyriaceae, unidentified family in order Pleosporales, Spiculogloeaceae, Rhynchogastremataceae, and Mycosphaerellaceae were more abundant in resistant lines, while Phyllachoraceae, Didymosphaeriaceae, Massarinceae, and Leptosphaeriaceae were more prevalent in susceptible lines (Figure 6A). At the genus level, *Cladosporium, Coniothyrium, Alternaria, Epicoccum, Bipolaris,* unknown genus from Pleosporales*, Phyllozyma, Papiliotrema, Cercospora, Stagonospora* and *Symmetrospora* were notably most abundant in resistant lines, whereas *Phyllachora*, *Paraphaeosphaeria*, and *Sphaerellopsis* showed higher relative abundance in susceptible lines (Figure 6B). Interestingly, family Phyllachoraceae and genus *Phyllachora* were detected in all inbreds. Numbers of *Phyllachora* reads mostly corresponded to the disease score exhibited by the inbred lines, with PI685920, B97, PI685788, 4401350, PI685918, PI685836, PI685950, PI685919, PI685790, and PI685915 having more than 30% *Phyllachora* reads and PI685806, PI685831, CML69 and CML103 having reads lower than 30% (Figures 6A,B and Table 1). In contrast, *Phyllachora* read percents in inbred lines PI685950, PI685919, PI685788, PI685790 and B97 were not consistent with the visual disease assessment (Table 1). Furthermore, the family Leptosphaeriaceae was most abundant on the inbred lines PI685915 and B97 whereas family Didymosphaeriaceae was most abundant on susceptible inbred lines PI685836, PI685918, 4,401,350 and PI685788 (Figure 6A). Interestingly, the family Massarinaceae was detected only on PI685836 and PI685918. At the genus level, *Fusarium* was predominant only on the line PI685831, *Bipolaris* was the most-abundant genus on the line PI685915, whereas *Sphaerellopsis* was most abundant on inbreds PI685915, and B97 (Figure 6B). The fungal phyla Ascomycota and Basidiomycota were the dominant fungi on all inbred lines (Supplementary Figure S5). These findings indicate that the phyllosphere microbiome of the fourteen inbred corn lines evaluated had low diversity in terms of bacterial and fungal distribution, with only a few dominant taxa identified. Although sample-to-sample variability is still high, the distribution of dominant bacterial and fungal taxa in all inbreds followed the same basic trend.

### 3.4 Identification of differential taxon abundance between resistant and susceptible lines

**Figure 7:**
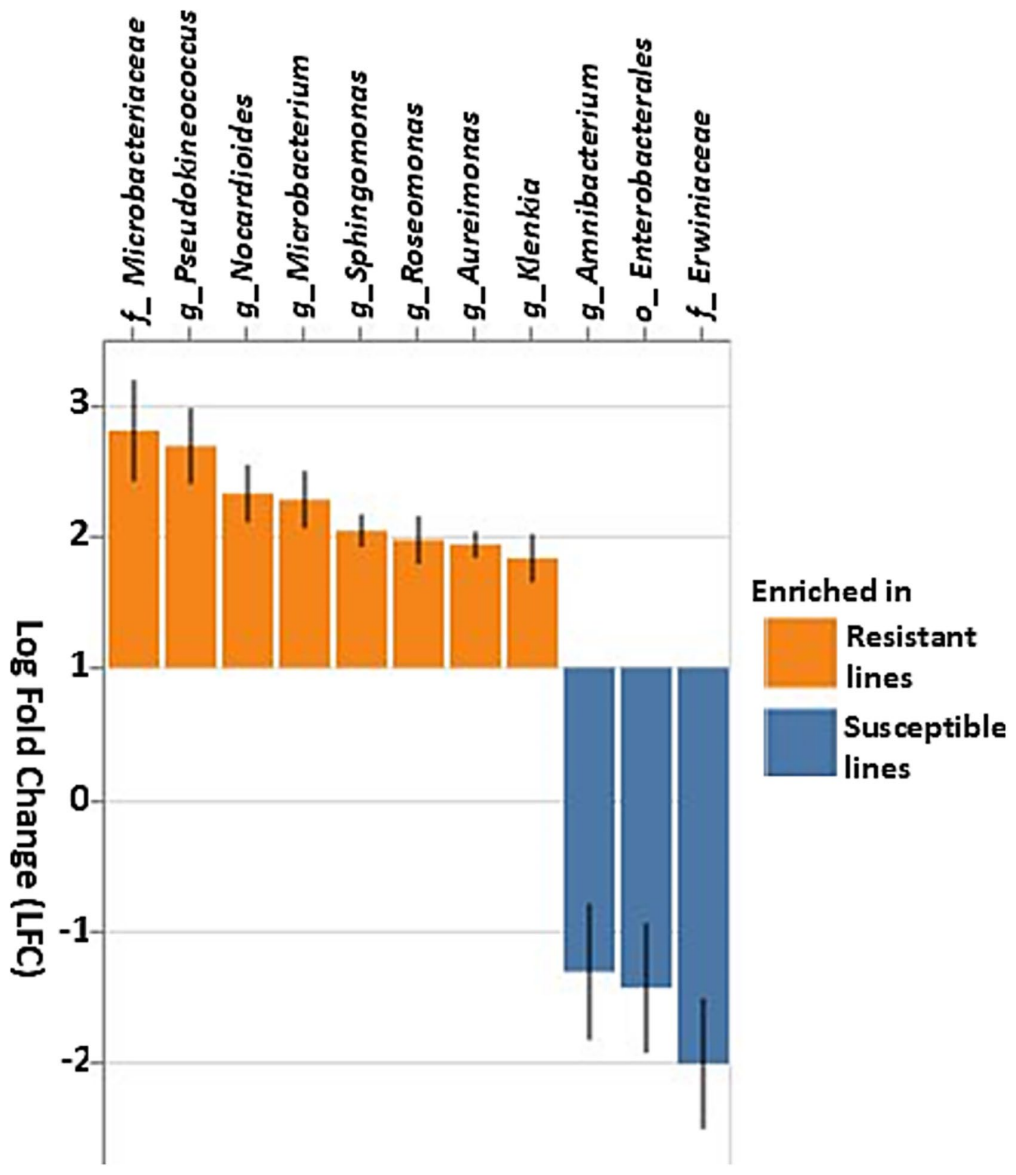
Differential abundance of bacterial taxa based on the ANCOM-BC (Analysis of composition of microbiomes analysis with bias correction) in resistant and susceptible corn lines. Taxa that differ significantly between the resistant and susceptible lines are displayed with log fold change (LFC) and 95% confidence error bars. Taxa showing positive LFC values were enriched in the resistant lines, whereas taxa with negative LFC values were enriched in the susceptible lines. The threshold for significance was set to a Benjamini–Hochberg false discovery rate-corrected *p*-value of 0.001.

Differential abundance analysis using ANCOM-BC revealed significant differences in the relative abundance of several key bacterial taxa between resistant and susceptible lines (Figure 7). The family Erwiniaceae and the order Enterobacterales, particularly the genus *Amnibacterium*, were significantly enriched in susceptible lines. Conversely, multiple genera within the family Microbacteriaceae, including *Klenkia*, *Aureimonas*, *Roseomonas*, *Sphingomonas*, *Microbacterium*, *Nocardioides*, and *Pseudokineococcus*, were the most abundant prokaryotic taxa in the resistant inbred lines (Figure 7).

**Figure 8:**
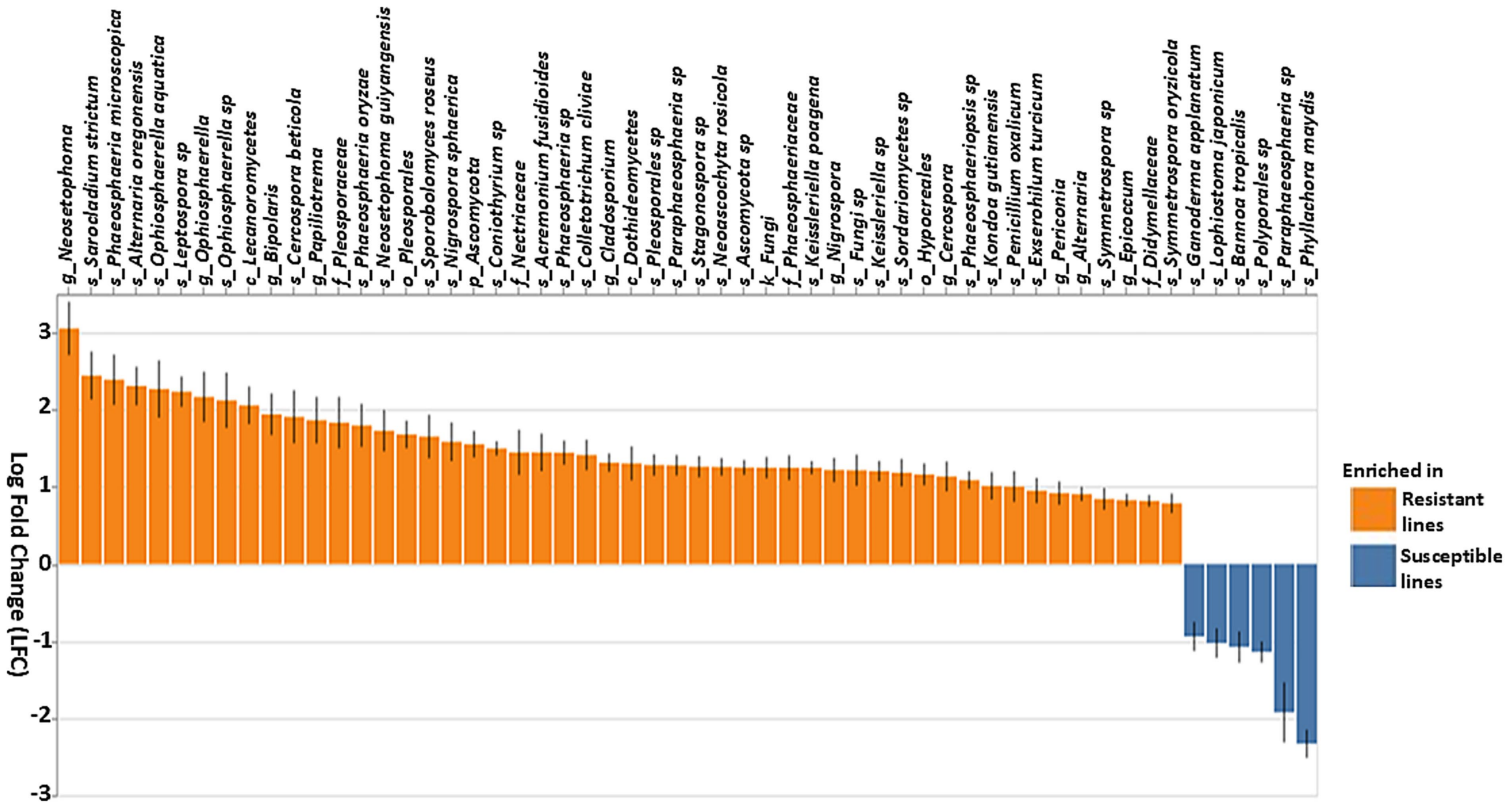
Differential abundance of fungal taxa based on the ANCOM-BC (Analysis of composition of microbiomes analysis with bias correction) in resistant and susceptible lines. Genera that differentiate significantly between the resistant and susceptible lines are displayed with log fold change and 95% confidence error bars. Taxa showing positive LFC values were enriched in the resistant lines, whereas taxa with negative LFC values were enriched in the susceptible lines. The threshold for significance was set to a Benjamini–Hochberg false discovery rate-corrected *p*-value of 0.001.

Additionally, fungal community analysis using ANCOM-BC identified several fungal genera that were differentially abundant between resistant and susceptible lines (Figure 8). Several Ascomycota, including *Neosetophoma, Sarocladium strictum, Phaeosphaeria microscopica, Alternaria oregonensis, Oohiosphaerella aquatica, Leptospora* sp.*, Bipolaris, Papiliotrema, Sporobolomyces roseus, Nigrospora sphaerica*, and *Coniothyrium* sp. showed marked enrichment in resistant lines (Figure 8). In contrast, several other species, such as *Ganoderma applanatum, Lophiostoma japonicum*, and *Bannoa tropicalis*, were more abundant in susceptible lines. Overall, these results demonstrate that both bacterial and fungal communities are distinct in resistant compared to susceptible lines, suggesting a complex interplay of microbial taxa associated with the observed phenotypes.

### 3.5 Tar spot disease severity level altered the phyllosphere microbiome

*Phyllachora maydis* reads were observed in all inbred lines irrespective of their severity level. To highlight potential links between the bacterial and fungal taxa with increasing *P. maydis* reads, Pearson correlation coefficients were calculated between the relative abundances of the 20 most abundant bacterial (Table 3) and fungal (Table 4) taxa. Positive correlations suggest that species tend to co-occur within the same sample and their abundances tend to increase or decrease simultaneously. Conversely, negative correlations indicate that an increase in one species’ abundance within a sample is associated with a decrease in another’s. Intriguingly, correlation analysis revealed a predominance of negative correlations, at least for the most abundant taxa in the bacterial and fungal communities, suggesting a tendency toward mutual exclusivity or an inverse relationship in their occurrence.

**Table 3:** The 20 most common bacterial taxa over all inbred lines and replications identified to most likely taxon, and their correlations with the percent of *Phyllachora maydis* reads.

Taxon	Pearson correlation	Accessions	Family	Order	Class	Phylum	Likely niche
*Methylobacterium komagatae*	−0.287***	GU046508.1	Beijerinckiaceae	Rhizobiales	Alphaproteobacteria	Pseudomonadota	Methylotrophic
*Sphingomonas* sp.	−0.358***	MH061269.1	Sphingomonadaceae	Sphingomonadales	Alphaproteobacteria	Pseudomonadota	Plant pathogen; biocontrol^1^
*Pantoea ananatis*	0.335***	LC015551.1	Erwiniaceae	Enterobacterales	Gammaproteobacteria	Pseudomonadota	Saprobe, opportunistic animal pathogen
*Quadrisphaera* sp.^2^	−0.374***	MT911353.1	Kineosporiaceae	Kineosporiales	Actinobacteria	Actinobacteriota	Unknown
*Klenkia terrae*	−0.348***	NR_109441.1	Geodermatophilaceae	Frankiales	Actinobacteria	Actinobacteriota	Saprobe
*Spirosoma radiotolerans*	−0.258***	CP010429.1	Spirosomaceae	Cytophagales	Bacteroidia	Bacteroidota	Unknown
*Nocardioides lentus*	−0.345***	NR_043566.2	Nocardioidaceae	Propionibacteriales	Actinobacteria	Actinobacteriota	Saprobe
*Hymenobacter*	−0.150***		Hymenobacteraceae	Cytophagales	Bacteroidia	Bacteroidota	Unknown
*Aureimonas pseudogalii*	−0.259***	KT806080.2	Aurantimonadaceae	Hyphomicrobiales	Alphaproteobacteria	Pseudomonadota	Unknown
*Pseudomonas syringae*	0.141**	PQ614806.1	Pseudomonadaceae	Pseudomonadales	Gammaproteobacteria	Pseudomonadota	Saprophyte; biocontrol^3^
*Microbacteriaceae bacterium*	−0.149***	OL444838.2	Microbacteriaceae	Micrococcales	Actinobacteria	Actinobacteria	Saprobe
*Sphingomonas* sp.	−0.135***	KX056915.1	Sphingomonadaceae	Sphingomonadales	Alphaproteobacteria	Pseudomonadota	Saprobe
*Deinococcus citri^4^*	0.262*	LT602921.1	Deinococcaceae	Deinococcales	Deinococci	Deinococcota	Polyextremophile^5^; biocontrol
*Microbacterium zeae*	−0.326***	OR122058.1	Microbacteriaceae	Micrococcales	Actinobacteria	Actinobacteria	Endophyte
*Roseomonas* sp.	−0.182***	LT008700.1	Acetobacteraceae	Acetobacterales	Alphaproteobacteria	Proteobacteria	Aerobic
*Marmoricola* sp.	−0.293***	MN620397.1	Nocardioidaceae	Propionibacteriales	Actinobacteria	Actinobacteriota	Chemoorganotrophic
*Curtobacterium* sp.	−0.142	MH769454	Microbacteriaceae	Micrococcales	Actinomycetia	Actinomycetota	plant pathogen
*Nakamurella deserti*	0.124*	NR_180061.1	Nakamurellacese	Nakamurellales	Actinomycetia	Actinomycetota	Unknown
*Pseudokineococcus* sp.	−0.409***	KX274780.1	Kineosporiaceae	Kineosporiales	Actinobacteria	Actinobacteriota	Aerobic
*Sphingomonas* sp.	−0.112***	MT900599.2	Sphingomonadaceae	Sphingomonadales	Alphaproteobacteria	Pseudomonadota	Unknown

Negative correlations imply mutual exclusivity of the taxon and *P. maydis*. Asterisks indicate significant *p-*values at 0.05 (*), 0.01 (**), and 0.001 (***). ^1^Some species of *Sphingomonas* may enhance disease resistance in rice (Matsumoto et al., 2021). ^2^Previous study showed some species of *Quadrisphaera* such as *Quadrisphaera granulorum* was significantly enriched in the microbiome of healthy ginger crop (*Zingiber officinale* L. Roscoe) against rhizome rot (Wang et al., 2024). ^3^*Pseudomonas syringae* strain ESC-11 effectively controls several postharvest diseases, including blue mold (caused by *Penicillium expansum*), gray mold (caused by *Botrytis cinerea*), and Mucor rot (caused by *Mucor* spp.) on apple and pear (Janisiewicz and Jeffers, 1997; Janisiewicz and Marchi, 1992). It also controls blue mold (caused by *Penicillium italicum*) and green mold (caused by *Penicillium digitatum*) on citrus fruits. *P. syringae* ESC-11 and *P. syringae* ESC-10 are commercially available for the control of postharvest decays of pear and apple and citrus fruits, respectively. ^4^Causal agent of Citrus Leaf Canker (Ahmad et al., 2014). ^5^Can tolerate a range of environmental stresses including extreme temperatures, dessication, radiation, oxidation and vacuum. Some species of *Deinococcus* could be used in biocontrol of rice foliar pathogens (Yang et al., 2008).

**Table 4:** The 20 most abundant fungal species encountered over all inbreds.

Txon	Pearson correlation	Accessions	Family	Order	Class	Phylum	Likely niche
*Phyllachora maydis*	1.000	OP984079	Phyllachoraceae	Phyllachorales	Sordariomycetes	Ascomycota	Tar spot pathogen
*Cladosporium* sp.	−0.486***	MW764235	Cladosporiaceae	Capnodiales	Dothideomycetes	Ascomycota	Endophytes, pant pathogens
*Coniothyrium* sp.	−0.444***	MG182687	Coniothyriaceae	Pleosporales	Dothideomycetes	Ascomycota	Possible mycoparasite
*Alternaria alternata*	−0.505***	KT968762	Pleosporaceae	Pleosporales	Dothideomycetes	Ascomycota	Plant pathogen
*Bipolaris_zeicola^#^*	−0.161**	KX834939	Pleosporaceae	Pleosporales	Dothideomycetes	Ascomycota	Cause of Northern corn eaf blight^a^
*Epicoccum nigrum^##^*	−0.379***	MG602580	Didymellaceae	Pleosporales	Dothideomycetes	Ascomycota	Endophyte, plant pathogens, antifungal agents^b^
*Neosetophoma* sp	−0.376***	MT777372	Didymosphaeriaceae	Pleosporales	Dothideomycetes	Ascomycota	Saprobe
*Papiliotrema flavescens*	−0.571***	MK352071	Rhynchogastremataceae	Tremellales	Tremellomycetes	Basidiomycota	Saprobe; yeast^###^
*Phyllozyma linderae^&^*	−0.268***	NR_073319	Spiculogloeaceae	Spiculogloeales	Spiculogloeomycetes	Basidiomycota	Saprobe; yeast
*Didymocyrtis pini*	−0.223***	PQ158733	Phaeosphaeriaceae	Pleosporales	Dothideomycetes	Ascomycota	Lichenicolous fungus
*Didymella pomorum*	−0.407***	OP596100	Didymellaceae	Pleosporales	Dothideomycetes	Ascomycota	Plant parasite cause of leaf spots and stem canker
*Paraphaeosphaeria neglecta* ^$^	0.378***	MW497207	Didymosphaeriaceae	Pleosporales	Dothideomycetes	Ascomycota	Likely saprobe
*Hannaella zeae*	−0.153**	KY611821	Bulleribasidiaceae	Tremellales	Tremellomycetes	Basidiomycota	Saprobe; yeast^c^
*Cercospora zeae-maydis^d^*	−0.213***	OR945717	Mycosphaerellaceae	Mycosphaerellales	Dothideomycetes	Ascomycota	Cause of grey leaf spot
Fungi	−0.311***	OP419710	Fungi	Fungi	Fungi	Basidiomycota	Not applicable
*Sphaerellopsis ilum^e^*	0.234**	EF600974	Leptosphaeriaceae	Pleosporales	Dothideomycetes	Ascomycota	Mycoparasite of *Puccinia*
*Stagonospora* sp.	−0.334***	OM236799	Massarinaceae	Pleosporales	Dothideomycetes	Ascomycota	Plant pathogen
Unclassified Pleosporales	−0.455***	PQ158979	Didymosphaeriaceae	Pleosporales	Dothideomycetes	Ascomycota	Not applicable
*Symmetrospora symmetrica^f^*	−0.445***	OP045204	Symmetrosporaceae	Cystobasidiomycetes	Cystobasidiomycetes	Basidiomycota	Saprobe, Red-pigmented yeast^f^
*Phaeosphaeriopsis* sp.	−0.319***	MT138620	Phaeosphaeriaceae	Pleosporales	Dothideomycetes	Ascomycota	Possible plant pathogen or saprobe
*Bullera alba^g^*	−0.237***	AF444662	Bulleraceae	Tremellales	Tremellomycetes	Basidiomycota	Yeast

Count is the sum of read counts over all inbreds and replicates. Pearson’s correlation coefficient r was calculated from abundance of each taxon versus abundance of *Phyllachora maydis* in the same inbred and replicate. Negative correlations imply mutual exclusivity of the taxon and *P. maydis*. Asterisks indicate significant *p* values at 0.05 (*), 0.01 (**), and 0.001(***). ^a^Causes Northern Corn Leaf blight; sexual stage is *Cochliobolus carbonum*. ^b^*Epicoccum nigrum* exerts antagonistic effects on various plant pathogens (*Sclerotinia sclerotiorum, Pythium irregulare*, and *Monilinia* spp.) (Taguiam et al., 2021; Zhou and Reeleder, 1989; Koutb and Ali, 2010). ^&^ Synonym: *Sporobolomyces linderae*; recently renamed *Phyllozyma linderae*. ^$^Previously had been a member of *Paraconiothyrium sporulosum* complex (Verkley et al., 2014). ^c^Asexual stage is *Cryptococcus zeae*. ^d^Causes grey leaf spot on corn. Sexual stage: Bai,2004 *Mycosphaerella* sp. ^e^Sexual stage: *Eudarluca caricis*. Potential biological control against Rust Disease (Gómez-Zapata et al., 2024). ^f^Basionym: *Sporobolomyces symmetricus* (Bai and Wang, 2004; Wang and Bai, 2004). ^g^Basionym: *Sporobolomyces albus*; Synonyms: *Bulleromyces*; Sexual stage: *Bulleromyces albus*; *B. alba* produced the killer toxin named microcin which showed strong antifungal activity against *Cryptococcus neoformans* (Golubev et al., 1997). # *Bipolaris* sp. similar to *Bipolaris zeicola* is a potential biocontrol agent for *Microstegium vimineum* (Japanese stiltgrass) (Kleczewski and Flory, 2010).

## *Epicoccum nigrum* can be used as biological pathogen inhibitors to inhibit *Fusarium graminearum* infection (Li T. et al., 2022b). ### *P. flavescens* has been pursued as a biocontrol agent of *Fusarium graminearum* in wheat (Shude et al., 2022). The related species *P. terrestris* is a potential biocontrol agent for fungal diseases of apple (Palmieri et al., 2021). Volatile organic compounds produced by *P. flavescens* have been shown to increase systemic resistance in Arabidopsis (Liu et al., 2024). Synonym *Cryptococcus flavescens.*

Among the 20 most abundant bacterial taxa, only four species, *Pantoea ananatis* (Pearson correlation: 0.335, *p* < 0.001)*, Pseudomonas syringae* (Pearson correlation: 0.141, *p* < 0.01)*, Deinococcus citri* (Pearson correlation: 0.262, *p* < 0.05), and *Nakamurella deserti* (Pearson correlation: 0.124, *p* < 0.05), were statistically and positively correlated with *P. maydis* reads, while 16 bacterial taxa were significantly negatively correlated with *P. maydis* reads (Table 3). *Pseudokineococcus* sp. showed the strongest negative correlation (Pearson correlation: −0.409, *p* < 0.001). Other notable negatively correlated taxa included *Quadrisphaera* sp. (Pearson correlation: −0.374, *p* < 0.001)*, Nocardioides lentus* (Pearson correlation: −0.345, *p* < 0.001), *Microbacterium zeae* (Pearson correlation: −0.326, *p* < 0.001), *Methylobacterium komagatae* (Pearson correlation: −0.287, *p* < 0.001)*, Spirosoma radiotolerans* (Pearson correlation: −0.258, *p* < 0.001) and *Aureimonas pseudogalii* (Pearson correlation: −0.259, *p* < 0.001) (Table 3). Some taxa, such as *Sphingomonas* sp. and *Curtobacterium* sp., showed correlations with *P. maydis* but these were not statistically significant at the 0.05 level.

In addition, for fungal species, only *Paraphaeosphaeria neglecta* (Pearson correlation: 0.378, *p* < 0.001), and *Sphaerellopsis filum* (Pearson correlation: 0.234, *p* < 0.01), were positively correlated with *P. maydis* reads, suggesting a potential synergistic relationship or a shared preference for similar environmental conditions (Table 4). Both of these genera contain mycoparasites which would likely increase in proportion to their food source. *Papiliotrema flavescens* (Pearson correlation: −0.571, *p* < 0.001) showed the strongest negative correlation suggesting a potential antagonistic relationship or a strong preference for different ecological niches. Additionally, *Alternaria alternata* (Pearson correlation: −0.505, *p* < 0.001)*, Cladosporium* sp. (Pearson correlation: −0.486, *p* < 0.001), Unclassified Pleosporales (Pearson correlation: −0.455, *p* < 0.001)*, Symmetrospora symmetrica* (Pearson correlation: −0.445, *p* < 0.001)*, Coniothyrium* sp. (Pearson correlation: −0.444, *p* < 0.001)*, Didymella pomorum* (Pearson correlation: −0.407, *p* < 0.001)*, Epicoccum nigrum* (Pearson correlation: −0.379, *p* < 0.001)*, Neosetophoma* sp. (Pearson correlation: −0.376, *p* < 0.001)*, Stagonospora* sp. (Pearson correlation: −0.334, *p* < 0.001)*, Phaeosphaeriopsis* sp. (Pearson correlation: −0.319, *p* < 0.001), Fungi sp. (Pearson correlation: −0.311, *p* < 0.001), *Phyllozyma linderae* (Pearson correlation: −0.268, *p* < 0.001), *Bullera alba* (Pearson correlation: −0.237, *p* < 0.001), *Didymocyrtis pini* (Pearson correlation: −0.223, *p* < 0.001) and *Cercospora zeae-maydis* (Pearson correlation: −0.213, *p* < 0.001), were statistically and negatively correlated with *P. maydis* reads (Table 4). *Bipolaris zeicola* (Pearson correlation: −0.161, *p* < 0.01) and *Hannaella zeae* (Pearson correlation: −0.153, *p* < 0.01) also showed negative correlations with *P. maydis* reads but these were less statistically significant. The overall findings showing more negative correlations with *P. maydis* reads indicate a high possibility for increased ecologic niche differentiation because of a high proportion of competition among bacterial and fungal communities.

In addition to tar spot, the sequence data also indicated corn lines that were susceptible to other common fungal pathogens. For example, inbreds PI685831, PI685915 and PI685806 were susceptible to northern corn leaf spot caused by *Bipolaris zeicola*, and line PI685920 to gray leaf spot (*Cercospora zeae-maydis*) (Figure 6B). Interestingly, the inbreds with high read numbers from *Phyllachora maydis* had low sequence reads for *Coniothyrium* sp., a possible mycoparasite of *Phyllachora maydis*. Therefore, the microbiome sequences provided a good overall picture of the fungi interacting with each other and their host substrate in the corn phyllosphere.

## 4 Discussion

The phyllosphere can contain a wide variety of beneficial, pathogenic, and commensal microbes (Lindow and Brandl, 2003). Although phyllospheric microbial communities in corn have been studied to some extent (Ferreira et al., 2025; McCoy et al., 2019; Singh and Goodwin, 2022), comparatively little is known about the structure, diversity, and community dynamics of this microbiome or its interactions with the host (Peñuelas and Terradas, 2014; Singh and Goodwin, 2022). Further analysis of the foliar microbiome is critical for understanding the types of pathogens present and evaluating the potential of other microbes to predict or prevent disease progression.

To test the hypothesis that microbiome composition varies in response to *P. maydis* infection in resistant and susceptible lines, we sequenced rRNA amplicons to investigate bacterial and fungal populations in 16 corn inbreds that varied in tar spot severity. Our findings revealed that fungal taxa and populations exhibited a more pronounced response to increasing *P. maydis* sequence counts compared to bacteria.

### 4.1 Impact of *P. maydis* infection on microbial alpha diversity

Alpha-diversity indices for bacterial and fungal microbiomes differ between resistant and most of the susceptible corn inbreds. For bacterial communities, we found no significant variation in species richness or evenness between resistant and most susceptible inbreds, suggesting similar species richness and similar distribution of species among these lines (Figures 1A,B). However, susceptible inbreds B97 and PI685788 were exceptions, with significantly lower Shannon diversities than the resistant lines, suggesting presence of a less diverse community with either fewer species or an uneven abundance of species in these lines (Figure 1A). While Faith’s phylogenetic diversity of bacterial communities was initially observed to be lower in the most susceptible lines compared to the two resistant lines (CML103 and CML69), this difference was not statistically significant across all lines tested (Figure 1C). This suggests that while there might be a trend toward lower phylogenetic diversity in susceptible lines, the evidence is not strong enough to draw a definitive conclusion. The lack of clear and consistent patterns across all alpha diversity metrics for bacterial communities suggests the possibility that specific bacterial taxa, rather than overall diversity, are crucial for influencing disease status, or that bacterial communities interact with other factors, such as the fungal microbiome or the host’s immune system, or the environmental factors in a more complex manner.

In contrast, fungal alpha diversity metrics showed a significant difference in fungal community diversity between resistant and susceptible lines (Figures 2A–C), a pattern not consistently observed for bacterial diversity (Figures 1A–C). Higher species richness was observed in most of the resistant lines compared to susceptible inbreds, suggesting that tar spot disease severity (excessive pathogen load) has a negative impact on bacterial and fungal community richness (Figures 1, 2). This also can be explained by the possibility that beneficial microbes may lose their selective advantage when pathogen load is excessive. Our findings align with a previous study on Southern leaf blight-susceptible plants, which also showed decreased leaf epiphytic bacterial species richness (Manching et al., 2014).

We did not see a clear difference in bacterial community diversities between the resistant and susceptible lines (Figures 1A,B). Except for the susceptible inbred lines B97 and PI685788, which were significantly lower, the other seven susceptible lines showed similar levels of bacterial community diversities (Figures 1A,B). The overlap in diversity metrices between resistant and susceptible lines, along with the presence of susceptible lines with low diversity, suggests that diversity alone is not the sole determinant of resistance. Other factors, such as specific microbial taxa, functional capabilities, or host genetics, likely play important roles. The higher Faith’s phylogenetic diversity in resistant lines, especially CML103 and CML69, indicates that a more diverse and phylogenetically rich community may be associated with enhanced resistance (Figure 1C). However, there was a significant difference in fungal community diversity between resistant and susceptible lines depending on the sample; the ten most-susceptible lines (PI685915, PI685790, PI685919, PI685950, PI685836, PI685918, B97, 4401350, PI685788, and PI685920) had significantly lower fungal community diversities compared to those of the resistant lines CML103, CML69, PI685806, and PI685831 (Figures 2A–C). These findings suggest that *P. maydis* infection might have a significant impact primarily on the richness of the phyllosphere microbiome and its fungal diversity, but not on bacterial diversity when including evenness.

### 4.2 Distinct microbial community structures (beta diversity) in resistant and susceptible maize

Both Bray-Curtis and Unweighted UniFrac analyses demonstrated distinct clustering patterns for both bacterial and fungal communities in resistant and susceptible maize inbred lines (Figures 3, 4). Fungal communities showed a strong association with disease resistance, particularly in terms of relative abundance, as evidenced by the clear separation in the Bray-Curtis ordination and the high percentage of variation explained. In contrast, bacterial communities show a much weaker association, with substantial overlap between resistant and susceptible lines in both ordinations. The strong correlation between fungal community structure, particularly relative abundance, and resistance suggests that specific fungal taxa may be key mediators of the resistance phenotype. The weaker association observed for bacterial communities suggests a more complex interplay with resistance, potentially involving specific taxa or functional groups not captured by overall diversity measures. Further research is needed to identify the specific taxa driving the observed patterns and to elucidate their functional roles in resistance. A deeper understanding of these complex interactions could lead to the development of novel microbiome-based strategies for enhancing disease resistance in maize (Singh and Goodwin, 2022).

Our experiment was designed to compare the corn phyllospheric microbiomes between resistant and susceptible inbred lines after natural infection with *P. maydis*. Relying on natural infection was necessary because *P. maydis* has not been successfully cultured *in vitro* and appears to be obligately biotrophic. Although the life cycle of *P. maydis* is not known completely, it has been shown to overwinter on corn residue, and spores can be dispersed by rain splash or wind (Groves et al., 2020; Kleczewski et al., 2019; Parbery, 1963). The field plot for this experiment is only 14 miles from Lake Michigan and has an excellent environment for the development of tar spot (Telenko et al., 2020). The field plot had been heavily infected with *P. maydis* during the previous year (Telenko et al., 2020), and the conditions yielded a consistent difference in disease severity between resistant and susceptible lines (Table 1). Consistent with our results, a previous study identified a strong correlation between disease resistance and the phyllosphere microbiome of sorghum (*Sorghum bicolor*) after natural infection (Masenya et al., 2021) in South Africa.

### 4.3 Key bacterial taxa associated with resistance and susceptibility

The most abundant bacterial and fungal taxa fell into three likely categories: beneficial, pathogenic, or commensal. Although amplification yielded fewer bacterial reads than fungal, this is not proof that fungal cells outnumbered bacterial cells. Fungi have more rRNA genes per genome, and the fungal and bacterial rDNA fragments were amplified in separate reactions with different primers. Bacterial taxa in both resistant and susceptible lines were dominated by potentially plant-beneficial families Beijerinckiaceae, Erwiniaceae and Sphingomonadaceae, and genera like *Methylobacterium*, unknown genus from Erwiniaceae, *Sphingomonas* and *Quadrisphaera*. This is consistent with the finding of Wallace et al. (2018) that the vast majority of bacterial reads in corn microbiomes came from only two groups: Sphingomonads and the Methylobacteria. However, specific bacterial genera showed differential abundances. Resistant lines were enriched with genera such as *Sphingomonas, Quadrisphaera, Klenkia, Nocardioides, Aureimonas, Microbacterium, Roseomonas,* and *Pseudokineococcus* (Figures 5, 7). These beneficial genera promote stress tolerance and potentially improve overall plant growth, as illustrated by the following examples. *Nocardioides thermolilacinus*, a potential biocontrol agent in tomato, reduces disease severity due to foliar fungal pathogens *Alternaria solani* and *Corynespora cassiicola*, the oomycete *Phytophthora infestans*, and the bacterial pathogens *Pseudomonas syringae pv. tomato* and *Xanthomonas campestris* pv*. vesicatoria*, in both laboratory and field trials (Filho et al., 2008; Carrer Filho et al., 2008). Some species of *Sphingomonas* have been reported to enhance disease resistance in rice (Matsumoto et al., 2021). Additionally, some species of *Quadrisphaera* are enriched in the microbiome of healthy plants and may offer protection against soilborne pathogens (Wang et al., 2024). Isolates of *Nocardioides* promoted stress tolerance and exhibited antimicrobial activity against a variety of pathogens (Jiang et al., 2018; Sun et al., 2015) and may be of interest for controlling tar spot of corn.

In contrast, some bacteria promote plant disease directly as pathogens or indirectly by enhancing susceptibility to other pathogens. *Methylobacterium*, unknown genus of Erwiniaceae, *Pseudomonas, Deinococcus*, and *Amnibacterium* were elevated in the susceptible lines (Figures 5B, 7). Erwiniaceae and *Pseudomonas* include opportunistic pathogens that have been reported to cause disease in corn (Hinton and Bacon, 1995; Roper, 2011; Vidaver and Carlson, 1978). Abundant genera *Methylobacterium*, and *Sphingomonas* and *Klenkia* were associated with both resistant and susceptible lines, and they have been frequently observed in the phyllosphere of corn.

It is important to acknowledge the taxonomic limitations we faced. Some microbes were identified only as an “unknown genus” because they were either novel or unrepresented in current reference databases. This lack of resolution can obscure important biological relationships and presents several challenges for drawing definitive conclusions. For example, it can lead to an underestimation of the true community diversity and an incomplete understanding of microbial functions. Therefore, future research must incorporate more comprehensive sequencing techniques and expanded databases to bridge this knowledge gap.

### 4.4 Key fungal taxa associated with resistance and susceptibility

Fungal microbial communities were dominated by the families Phyllachoraceae, Cladosporiaceae, Pleosporaceae, and Didymellaceae and the genera *Phyllachora*, *Cladosporium*, *Coniothyrium* and *Alternaria*. *Phyllachora* reads were detected in all inbred lines, even those that were rated the most resistant, and disease severity generally increased with increasing percentage of *Phyllachora* sequences among fungal reads. Exceptions were inbreds PI685919, PI685790, PI685950, PI685788, and B97, which were scored as resistant to moderately resistant based on disease phenotype with ratings in the range of 0.0 to 5% for resistant and >5 to 11% for moderately resistant, but had very high (69 to 90%) *Phyllachora* sequence reads and thus were considered as susceptible for our analyses. This highlights the importance of considering both phenotypic and molecular data when assessing disease resistance and underscores potential limitations in both phenotypic and molecular detection methods due to factors such as scoring error or read quality. This finding is supported by a recent study that also identified *Phyllachora* reads in asymptomatic tissue with no visible symptoms (Ferreira et al., 2025).

If the lack of symptoms with relatively high *P. maydis* reads is not a reflection of experimental error, it suggests that stroma development can be suppressed in some corn genotypes or microbial environments that otherwise may permit infection. The most-abundant fungal genera in the resistant lines were *Cladosporium*, *Coniothyrium, Alternaria, Papiliotrema*, *Epicoccum, Bipolaris, Phyllozyma*, Pleosporales genus, Didymellaceae genus, *Cercospora, Stagonospora,* and *Symmetrospora.* These genera also predominated in resistant lines and have been found to be abundant in symptomatic leaves in other studies of tar spot infection (Ferreira et al., 2025; Inácio et al., 2002; McCoy et al., 2019; Singh and Goodwin, 2022; Wagner et al., 2019). Other possible explanations for why some lines showed few visible symptoms but had high *Phyllachora* reads could be that those were latent infections that would show symptoms later, or that the inbreds are tolerant to *P. maydis* so can support a relatively high level of infection with little disease. Additional experiments are required to test these alternative hypotheses.

Fungal genera such as *Cladosporium, Coniothyrium, Alternaria, Epicoccum, Bipolaris*, unknown genus from Pleosporales, *Phyllozyma, Papiliotrema, Cercospora, Acremonium, Stagonospora, Symmetrospora, Neosetophoma, Sarocladium strictum, Phaeosphaeria microscopica, Oohiosphaerella aquatica, Leptospora, Sporobolomyces roseus*, and *Nigrospora sphaerica* were more prevalent in resistant lines (Figures 6B, 8). Members of these genera have been demonstrated to exhibit biocontrol properties against various plant pests and diseases. For example, *Cladosporium* species suppressed whiteflies and aphids (Abdel-Baky and Abdel-Salam, 2003), *Bipolaris s*pecies exhibited a potential biocontrol against *Microstegium vimineum* (Kleczewski and Flory, 2010) and *Epicoccum nigrum* has antagonistic effects on *Sclerotinia sclerotiorum, Pythium irregulare, Monilinia* spp., and *Fusarium graminearum* (Taguiam et al., 2021; Zhou and Reeleder, 1989; Koutb and Ali, 2010; Li T. et al., 2022). Additionally, *Papiliotrema* spp. have controlled postharvest pathogens and fungal diseases of apple (Castoria et al., 2021; Palmieri et al., 2021) and *P. flavescens* has been pursued as a biocontrol agent of *Fusarium graminearum* in wheat (Shude et al., 2022). Volatile organic compounds produced by this species have been shown to increase systemic resistance in *Arabidopsis* (Liu et al., 2024), showing that various elements of microbiomes may affect plants through multiple mechanisms. These findings suggest that these fungal genera may be excellent candidates for further investigation into tar spot disease prevention. On the other hand, in addition to *Phyllachora* itself, *Paraphaeosphaeria, Sphaerellopsis, Polyporales* sp.*, Bannoa tropicalis, Lophiostoma japonicum, Ganoderma applanatum* and *Didymella* increased relatively in the susceptible inbreds, suggesting that these taxa may have contributed to tar spot susceptibility or disease progression (Figure 6B), or that they could be mycoparasites that increase in frequency with greater availability of their food source. The latter idea is supported by a study from Caldwell et al. (2024), which found *Paraphaeosphaeria* sp. only in tar spot samples. However, more investigation is required to confirm this hypothesis.

The composition and frequency of sequence reads provided an indication of other interactions between corn and its associated microbes. For example, although we did not score the inbreds for other diseases, lines that were susceptible could be identified by high numbers of reads for the pathogens that cause northern corn leaf spot (*Bipolaris zeicola*), and gray leaf spot (*Cercospora zeae-maydis* or *C. zeina*) (Figure 6B). Distribution of sequence reads also indicated more complex relationships. Reads from *Sphaerellopsis* were rare in inbreds that were resistant to tar spot but were common in two of the more susceptible lines (PI685915, and B97), the two lines that were clearly susceptible to rust. *Sphaerellopsis filum* is a known mycoparasite of *Puccinia* species (Gordon and Pfender, 2012; Trakunyingcharoen et al., 2014) and its frequency most clearly tracked that of its *Puccinia* hosts; it was virtually undetectable in lines that did not also seem to be susceptible to rust. While *Puccinia* was initially detected in rust-susceptible lines from raw reads, it was not detected in the same inbreds after quality filtering (Figure 2B). Our findings showed that most bacterial and fungal reads were mapped into only a few families and genera, in agreement with a previous study and confirming the relatively low diversity of the corn foliar microbiome (Wallace et al., 2018). While low diversity is consistent with leaf microbiome data from other species (Delmotte et al., 2009), it contrasts sharply with the high diversity generally found in the corn rhizosphere (Peiffer et al., 2013; Yang et al., 2017).

A diverse community could provide several benefits, such as increased competition for resources, a greater capacity to produce antimicrobial compounds, or a strong ability to induce host defense responses (Figure 2). Nevertheless, we cannot ignore the likelihood of host genetic influences and environmental effects on community diversity. Previously, it was shown that the genetic composition of the host structured the phyllosphere microbiome (da Silva et al., 2016; Wagner et al., 2019; Wallace et al., 2018). Additionally, corn recombinant inbred lines (RILs) revealed a major link between plant genetics, community diversity, and disease resistance (Balint-Kurti et al., 2010). Furthermore, high bacterial diversity in maize lines was linked to SLB susceptibility in the field (Balint-Kurti et al., 2010). Our findings contrast with those of Wallace et al. (2018), who found that corn genotype did not affect diversity of the foliar microbial community in an RIL population. Possibly the genetic diversity in the population analyzed by Wallace et al. (2018) was lower than those in our panel of inbreds or the RIL population analyzed by Balint-Kurti et al. (2010). It is also possible that environmental heterogeneity was great enough to obscure any genetic effects in the Wallace et al. (2018) study. The composition of the phyllosphere microbiome and the resulting disease status are significantly influenced by a range of environmental variables, such as temperature, humidity, and geographic location (Singh and Goodwin, 2022). Previous studies supported this, showing that the fungal microbiomes associated with tar spot vary significantly by geographic location (Caldwell et al., 2024; Sic-Hernandez et al., 2025). This indicates that regional environmental factors including climate, soil, and agricultural practice are powerful drivers of a microbiome’s composition.

The significant differentiation observed between the microbial communities of resistant and susceptible maize lines suggests a strong association between microbial composition and disease resistance as shown by beta diversity analysis. The distinct clustering patterns in both bacterial and fungal communities, as visualized by Bray-Curtis and Weighted Unifrac distances, underscore the unique microbial profiles of resistant and susceptible lines (Figures 3, 4). While some exceptions were noted, such as certain susceptible lines clustering with resistant lines, the overall trend indicates a clear divergence in microbial communities. Both Bray-Curtis and Weighted UniFrac analyses demonstrated distinct clustering patterns for both bacterial and fungal communities in resistant and susceptible maize lines with a stronger driver of separation due to phylogenetic differences (Figures 3, 4). This suggests that specific microbial taxa may play a role in conferring resistance to *Phyllachora maydis*.

### 4.5 Complex interactions and potential mechanisms driving microbiome shifts

Potentially beneficial bacterial and fungal taxa declined in susceptible lines in response to increased *P. maydis* load, implying that tar spot severity affects the bacterial and fungal communities. As the disease progresses, *P. maydis* might displace or inhibit these beneficial microbial taxa for its own advantage, further aggravating the disease. Previously, Manching et al. (2014) demonstrated that disease status shapes phyllospheric microbiome composition and structure. Meanwhile, other plant-pathogenic bacterial and fungal taxa increased in susceptible lines with increased *P. maydis* reads, indicating that these microbes might be associated with tar spot disease progression.

In general, host genotype, weather, availability of inoculum and other unknown factors may affect variation in the foliar microbiome. At least six specific relationships can operate within these limits:

1. *Phyllachora* invasion might physically displace a pre-existing microflora without changing its composition. In this case, reduced richness would reflect decreasing sample size of non-*Phyllachora* reads.
2. *Phyllachora* might compete with a co-existing microflora for water and nutrients from host cells, and the less-proficient competitors die out.
3. *Phyllachora* might produce soluble compounds, for example antibiotics, that directly antagonize other taxa.
4. *Phyllachora* might trigger host defense responses that differentially affect other organisms.
5. The corn inbreds vary in genotype for receptors that might allow *Phyllachora* to detect and attack host cells. Possibly these receptors also differentially affect other taxa.
6. Other organisms could chemically antagonize *Phyllachora*, and their antibacterials or antifungals could collaterally inhibit other microbes.

While physical displacement obviously occurs, our data suggest that one or more of the other mechanisms also might operate in tar spot disease.

### 4.6 Broader implications and future directions

To the best of our knowledge, this is the first analysis to investigate the bacterial and fungal microbiomes in the phyllospere of corn in relation to natural infection with *P. maydis* in both resistant and susceptible lines. We found that infection with *P. maydis* results in distinct microbial communities. Taxa that are differentially abundant between resistant and susceptible lines might be of interest for their potential in controlling tar spot of corn (Figures 7, 8). For example, susceptible lines were enriched in Erwiniaceae bacteria (especially *Amnibacterium*), while resistant lines showed greater abundance of Microbacteriaceae genera, suggesting potential roles in susceptibility and resistance, respectively. Resistant lines were also enriched in several Ascomycota fungi (e.g., *Neosetophoma, Fusarium, Alternaria*), possibly contributing to resistance, while susceptible lines showed greater abundance of other fungi (e.g., *Ganoderma, Lophiostoma, Bannoa*), potentially indicating a less robust environment against the pathogen. These distinct bacterial and fungal communities highlight a complex interplay of microbial taxa influencing tar spot development.

Correlation analysis revealed a complex relationship between *P. maydis* and the corn microbiome, with a predominance of negative correlations suggesting potential competition and niche differentiation within the microbial community (Tables 3, 4). Organisms with strong positive correlations with *Phyllachora* reads could be possible mycoparasites, which increase in abundance with greater availability of their food supply (Table 4). For example, *Sphaerellopsis filum,* which is positively correlated with *P. maydis* reads, is a known mycoparasite of rust fungi, suggesting it could potentially regulate other fungal populations in the maize microbiome (Gómez-Zapata et al., 2024). Additionally, *Paraphaeosphaeria neglecta,* which was positively correlated with *P. maydis*, is described as a “likely saprobe,” suggesting a potential synergistic relationship or a shared preference for similar environmental conditions (Verkley et al., 2014). Conversely, organisms that are highly negatively correlated with *Phyllachora* reads could be antagonists that compete directly or produce compounds that inhibit tar spot development (Table 4). Either group could be a source of organisms for possible biocontrol of *P. maydis*. For example, *Epicoccum nigrum,* which showed a negative correlation, is known to have antagonistic effects on various plant pathogens, supporting the hypothesis that *E. nigrum* may suppress *P. maydis* growth (Taguiam et al., 2021; Zhou and Reeleder, 1989; Koutb and Ali, 2010; Li T. et al., 2022). Additionally, *Alternaria alternata*, also negatively correlated, is a known mycoparasite, potentially acting as a natural control agent against other fungi, including *P. maydis* (Zheng et al., 2017) and *Papiliotrema flavescens*, showing the strongest negative correlation, is a known saprobe and yeast having antagonistic activity that could be due to competition for resources or the production of inhibitory compounds (Palmieri et al., 2021). This species also produces VOCs that can stimulate systemic resistance in Arabidopsis; if a similar response occurs in corn then it might make the plants more resistant to *P. maydis*. The basidiomycetous yeast *Bullera alba* produces a compound that has strong antifungal activity (Golubev et al., 1997), which may explain its negative correlation with *P. maydis* reads. Functional characterization of these potentially beneficial, differentially abundant taxa might elucidate important details of their interactions with *P. maydis* that could indicate how they might be deployed to mitigate tar spot disease in the future, such as the development of microbial-based biocontrol agents or the application of beneficial microbes to enhance plant health. The potential utility of these microbes for practical agricultural applications requires further validation through field-level experiments to support the development of sustainable practices.

The microbiome associated with tar spot has the potential to influence host defense and could be used for future microbiome-based management strategies. However, a significant gap remains in our understanding of microbial functionality, which is crucial for determining how microbes influence host health. This gap can be addressed using advanced approaches like metagenomics, metaproteomics, and metabolomics to gain deeper insights into microbial functions and connect them to host phenotypes (Guo et al., 2024). Recently, microbiome engineering has emerged as a promising strategy for directly manipulating functionally important microbes to improve overall plant fitness and productivity (Kaul et al., 2021; Batool et al., 2024). However, effective microbiome engineering requires a systems-level understanding of the corn microbiome, including microbe-microbe interaction networks and host–microbe interactions. This will allow us to successfully manipulate beneficial microbes (synthetic community) to improve plant resistance, fitness, and overall productivity.

#### Code availability

The source code for this study is available on GitHub and can be accessed at the following link (https://github.com/wilysic/Tar-spot-microbiome-and-corn-lines).

## Data Availability

The datasets presented in this study can be found in online repositories. The names of the repository/repositories and accession number(s) can be found in the article/Supplementary material.
