# Supplementary material for: Tar spot susceptibility of corn influences phyllosphere-associated bacterial and fungal microbiomes
Source: Front Microbiol. 2025 Oct 7;16:1581312. doi: 10.3389/fmicb.2025.1581312 (PMC12537778; doi:10.3389/fmicb.2025.1581312)
Supplement: Supplementary file 1 [file Table_1.docx]

| **Supplementary Table S1.** Total sequence reads, *Phyllachora maydis* sequence reads and percent *Phyllachora maydis* sequence reads for sixteen inbred lines of corn (*Zea mays* ssp. *maydis*) chosen from the parents of the Nested Association Mapping (NAM) and Germplasm Enhancement of Maize (GEM) genetic populations.   \| \| **Corn inbred line** \| **P. maydis reads** \| **Total assigned reads** \| \| --- \| --- \| --- \| \| PI685920 \| 445,109 \| 481,990 \| \| B97 \| 281,120 \| 315,066 \| \| PI685788 \| 368,618 \| 419,018 \| \| 4401350 \| 228,385 \| 262,956 \| \| PI685918 \| 364,582 \| 432,002 \| \| PI685836 \| 207,004 \| 258,744 \| \| PI685950 \| 167,864 \| 218,684 \| \| PI685919 \| 321,355 \| 435,672 \| \| PI685790 \| 334,208 \| 483,074 \| \| PI685915 \| 316,532 \| 481,556 \| \| PI685806 \| 136,603 \| 506,044 \| \| PI685831 \| 131,958 \| 564,142 \| \| CML69 \| 65,551 \| 435,572 \| \| CML103 \| 40,199 \| 428,250 \| \| CML52 \| - \| 86,852 \| \| TX303 \| - \| 19,308 \| \|  \|  \|  \| \| --- \| --- \| --- \| --- \| --- \| --- \| --- \| --- \| --- \| --- \| --- \| --- \| --- \| --- \| --- \| --- \| --- \| --- \| --- \| --- \| --- \| --- \| --- \| --- \| --- \| --- \| --- \| --- \| --- \| --- \| --- \| --- \| --- \| --- \| --- \| --- \| --- \| --- \| --- \| --- \| --- \| --- \| --- \| --- \| --- \| --- \| --- \| --- \| --- \| --- \| --- \| --- \| --- \| --- \| --- \| \|  \|  \|  \|  \| \|  \|  \|  \|  \| \|  \|  \|  \|  \| \|  \|  \|  \|  \| |  |  |  |  |
| --- | --- | --- | --- | --- | --- | --- | --- | --- | --- | --- | --- | --- | --- | --- | --- | --- | --- | --- | --- | --- | --- | --- | --- | --- | --- | --- | --- | --- | --- | --- | --- | --- | --- | --- | --- | --- | --- | --- | --- | --- | --- | --- | --- | --- | --- | --- | --- | --- | --- | --- | --- | --- | --- | --- | --- | --- | --- | --- | --- | --- | --- | --- | --- | --- | --- | --- | --- | --- | --- | --- | --- | --- | --- | --- | --- |
|  |  |  |  |  |
|  |  |  |  |  |
|  |  |  |  |  |
|  |  |  |  |  |
|  |  |  |  |  |
|  |  |  |  |  |
|  |  |  |  |  |
