# Supplementary figures and images for: Tar spot susceptibility of corn influences phyllosphere-associated bacterial and fungal microbiomes
Source: Front Microbiol. 2025 Oct 7;16:1581312. doi: 10.3389/fmicb.2025.1581312 (PMC12537778; doi:10.3389/fmicb.2025.1581312)

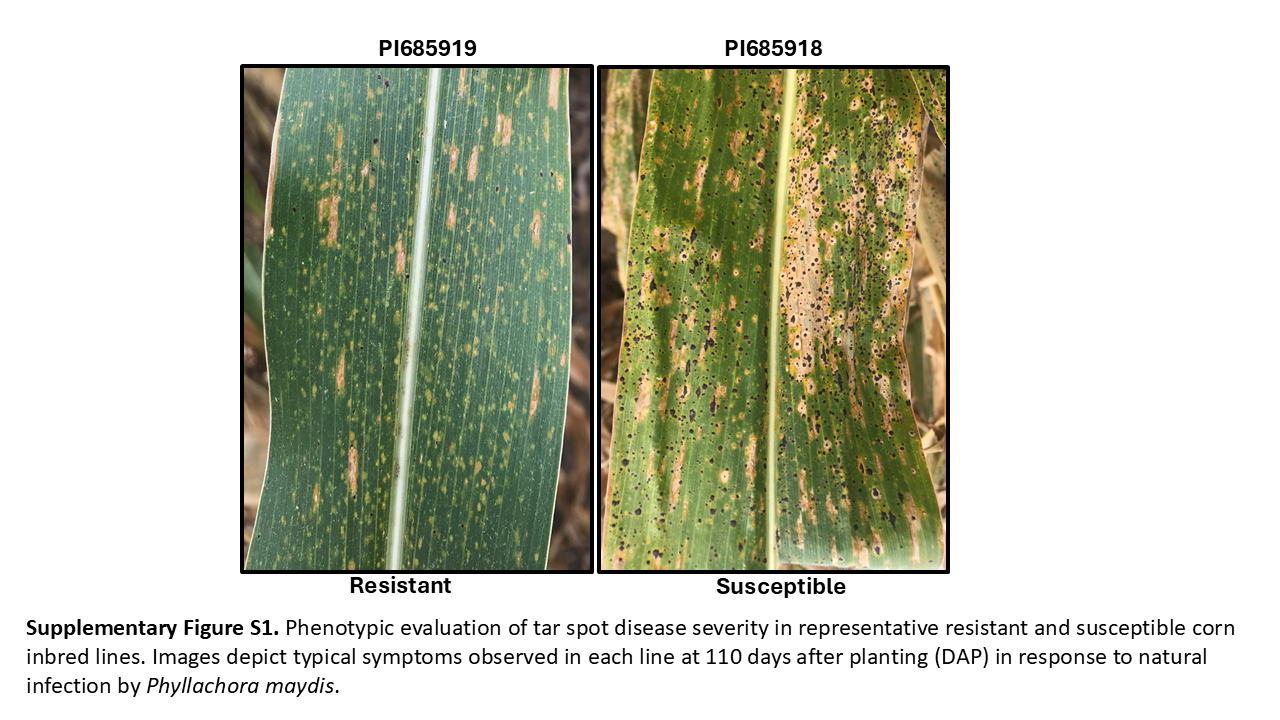

Supplement: Supplementary file 4 [file Image_1.tif]

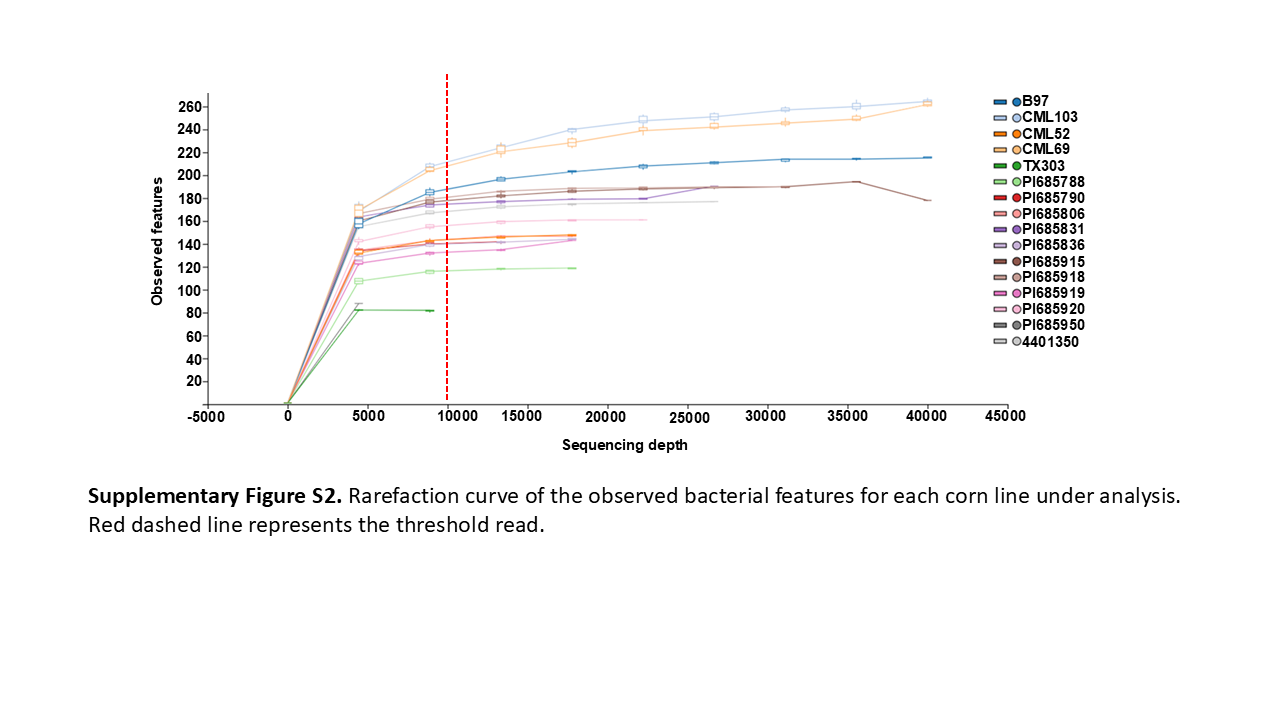

Supplement: Supplementary file 5 [file Image_2.tif]

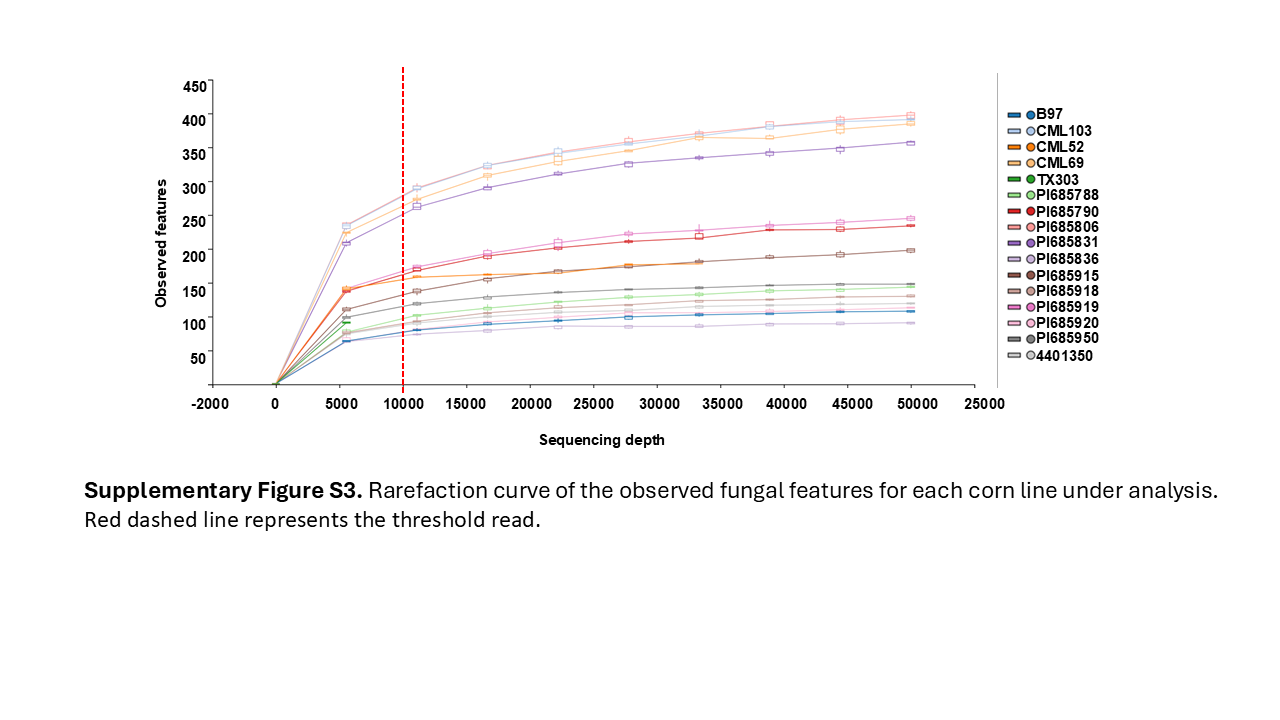

Supplement: Supplementary file 6 [file Image_3.tif]

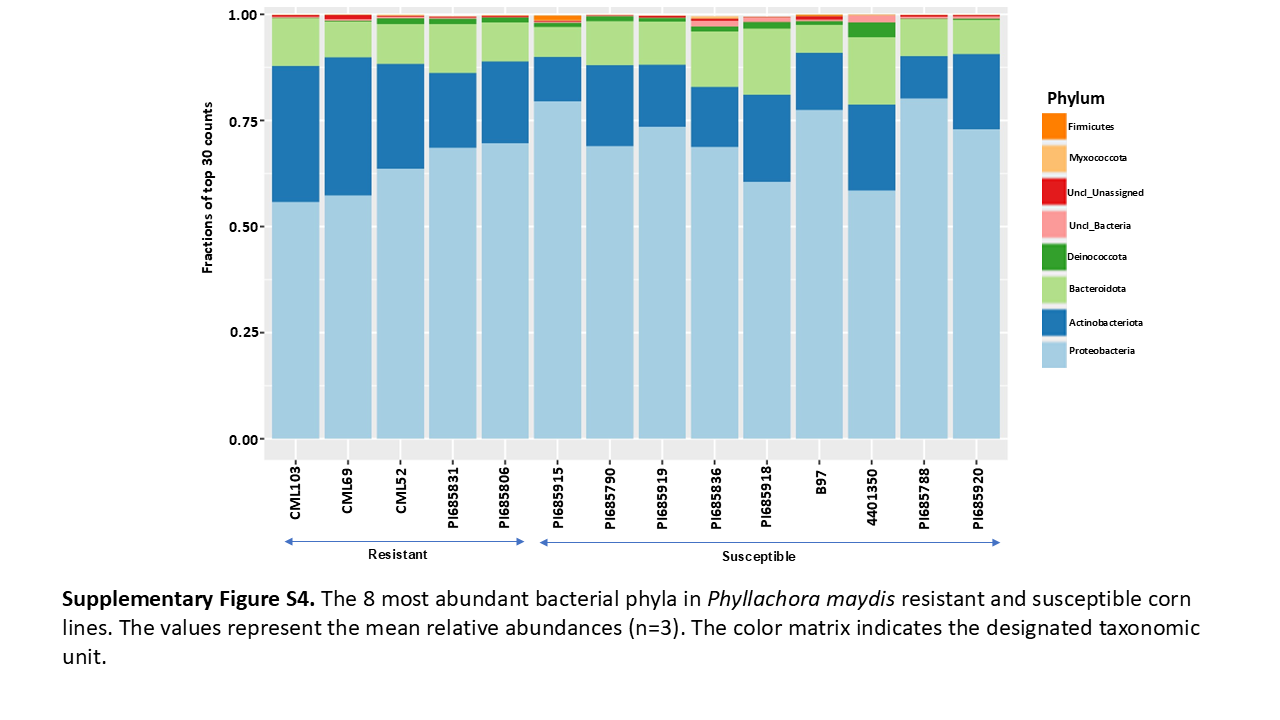

Supplement: Supplementary file 7 [file Image_4.tif]

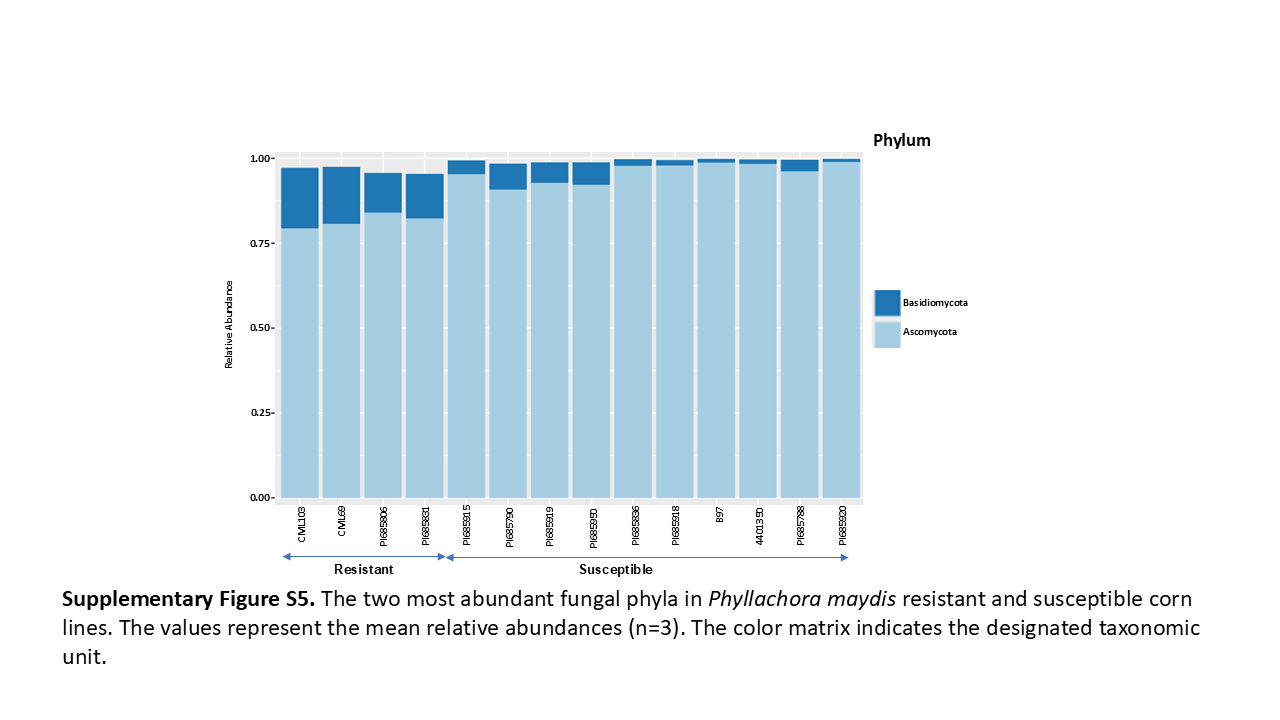

Supplement: Supplementary file 8 [file Image_5.tif]
